# Varying Responses to Heat Stress and Salinization Between Benthic and Pelagic Riverine Microbial Communities

**DOI:** 10.1111/1462-2920.70173

**Published:** 2025-09-04

**Authors:** Lisa Boden, Dana Bludau, Guido Sieber, Aman Deep, Daria Baikova, Gwendoline M. David, Una Hadžiomerović, Tom L. Stach, Dominik Buchner, Jens Boenigk

**Affiliations:** ^1^ Department Biodiversity University of Duisburg–Essen Essen Germany; ^2^ Centre for Water and Environmental Research University of Duisburg–Essen Essen Germany; ^3^ Department of Engineering and Natural Sciences Westphalian University of Applied Sciences Recklinghausen Germany; ^4^ Department Environmental Microbiology and Biotechnology University of Duisburg–Essen Essen Germany; ^5^ Department of Plankton and Microbial Ecology Leibniz Institute of Freshwater Ecology and Inland Fisheries (IGB) Stechlin Germany; ^6^ Environmental Metagenomics Research Center One Health Ruhr, University Alliance Ruhr University of Duisburg–Essen Essen Germany; ^7^ Department Aquatic Ecosystem Research University of Duisburg–Essen Essen Germany

**Keywords:** anthropogenic stressors, climate change, freshwater, heatwaves, mesocosm experiment, microbial communities, salinization

## Abstract

Microbial communities play a crucial role in the functioning of freshwater ecosystems but are continuously threatened by climate change and anthropogenic activities. Elevated temperatures and salinisation are particularly challenging for freshwater habitats, but little is known about how microbial communities respond to the simultaneous exposure to these stressors. Here, we use mesocosm experiments and amplicon sequencing data to investigate the responses of pelagic and benthic microbial communities to temperature and salinity increases, both individually and in combination. Our results highlight the varying responses of freshwater microbial communities, with sediment communities exhibiting greater stability in response to environmental changes compared to water column communities, and salinisation having a more pronounced impact on microeukaryotes compared to prokaryotes. Simultaneous exposure to elevated temperature and salinity reduced the impact of salinisation on prokaryotes, while microeukaryotes were similarly affected by the combined treatments and salinisation alone. These findings emphasise the complexity of microbial responses to single and multiple stressors, underscoring the need to consider both individual and interactive effects when predicting ecosystem responses to environmental changes.

## Graphical abstract

Mesocosm experiments reveal distinct responses of freshwater microbial communities to environmental change. Benthic communities remained more stable than those in the water column, while salinisation affected microeukaryotes more than prokaryotes. Elevated temperature mitigated salinisation effects on prokaryotes, emphasising the complexity of microbial responses to single and combined stressors.

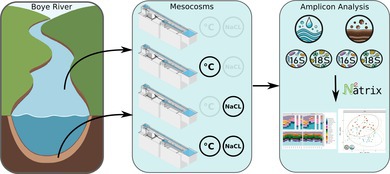

## 1 Introduction

Freshwater ecosystems are facing major challenges due to the combined effects of climate change and anthropogenic activities (Reid et al. 2019). As surface temperatures rise globally and human populations continue to grow, these environments are increasingly subjected to habitat degradation, pollution, and altered hydrological patterns (Dudgeon et al. 2006; Amoatey and Baawain 2019; Gunawardana et al. 2020). These changes threaten biodiversity (Dudgeon et al. 2006; Tickner et al. 2020) and significantly alter microbial community composition (Abdullah Al et al. 2021; Xie et al. 2021; Engloner et al. 2023) in freshwater habitats. Microbial communities play essential roles in nutrient cycling (Falkowski et al. 1998; Falkowski et al. 2008; Linz et al. 2018) and significantly impact water quality in natural habitats (Garrido et al. 2014; Zhao et al. 2020). Understanding how these communities respond to environmental changes is crucial, as community shifts can have cascading effects on ecosystem functioning and stability (Beattie et al. 2020; Martin et al. 2020; Mo et al. 2021).

Freshwater habitats face particular challenges from heat stress and salinization, both on their own and when they occur together (Sun and Arnott 2022). Climate change is steadily raising global surface temperatures and causing more frequent and intense heatwaves, during which surface water temperatures are expected to rise by 4°C–6°C (Woolway et al. 2021; Woolway et al. 2022; IPCC 2021). The magnitude and duration of these increases depend on the characteristics of the waterbody, such as depth, stratification, and degree of enclosure, as well as the duration of the heatwave, which can range from days to several weeks or even months (Woolway et al. 2021). These rising temperatures can significantly alter microbial community composition (Hao et al. 2018; Rocca et al. 2022; Engloner et al. 2023), primary production (Velthuis et al. 2017; Courcoul et al. 2022), and metabolic rates of bacteria (Yuan et al. 2020; Rocca et al. 2022) in aquatic ecosystems. Similarly, salinization of rivers and lakes due to human activities, such as agriculture, urbanisation, mining, and the use of de‐icing salts (Cañedo‐Argüelles et al. 2019), can cause significant shifts in taxon composition among both prokaryotic (Zhang et al. 2014; DeVilbiss et al. 2022; Gagnon et al. 2022) and microeukaryotic communities (Li et al. 2018; Fournier et al. 2021; Astorg et al. 2022), thereby significantly impacting carbon‐nutrient cycles (Duan and Kaushal 2015; Herbert et al. 2015; Sauer et al. 2016). In natural rivers, chloride (Cl^−^) concentrations typically fall between 1 and 20 mg·L^−1^ but can rise to 200 mg·L^−1^ in urban streams (Hintz and Relyea 2019). Even higher concentrations have been recorded, with mining effluent containing up to 3 g·L^−1^ (Gombert et al. 2019) and freshwater bodies affected by winter road salting showing levels as high as 8 g·L^−1^ (Hintz and Relyea 2019).

River water and sediment harbour distinct microbial communities, which vary in their ecological functions (Lu et al. 2016; Li et al. 2023; Wu, Zhao, et al. 2023) and are shaped by different abiotic factors (Cai et al. 2020; Li et al. 2023; Wu, Zhao, et al. 2023). Consequently, microbial community responses to environmental changes can significantly differ in water and sediment (Liao et al. 2019; Chang et al. 2020; Cai et al. 2020). Both heat stress and salinisation have been shown to have more pronounced effects on the prokaryotic community in water than in sediment (Tammert et al. 2023; Niu et al. 2024). Increases in temperature and salinity have also been shown to impact pelagic protist communities (Zou et al. 2021), while the impact of abiotic factors on microeukaryotic communities seems to be generally less significant in sediments (Shi et al. 2020; Dai et al. 2021). However, to our knowledge, no studies have directly compared the impact of heat stress and/or salinisation on pelagic and benthic microeukaryotic communities.

While both prokaryotic and microeukaryotes can be affected by heat stress and salinisation, particularly in the water column, sensitivity to abiotic stressors seems to vary between these two microbial groups. For example, bacteria are often more sensitive to temperature increases (Fang et al. 2021; Thakur et al. 2021) but seem to be more resistant than microeukaryotes to elevated salt concentrations (Gagnon et al. 2022). Nonetheless, little is known about how freshwater microbial communities respond to the simultaneous exposure to these two stressors (Velasco et al. 2018). Farias et al. (2024) suggest that increased salinity may reduce temperature tolerance in freshwater eukaryotes, but this study focused on algae and macroorganisms rather than microbial communities. To our knowledge, despite multiple previous studies on the responses of microorganisms to various stressor combinations (Burdon et al. 2020; Niu et al. 2024; Rocha et al. 2024), the combined effects of heat stress and salinisation on freshwater microeukaryotes remain largely unexplored, and no studies have investigated the response of prokaryotes.

Here, we conducted indoor mesocosm experiments to examine the individual and combined effects of a 5°C temperature increase and an increase in sodium chloride concentration of 2.5 g·L^−1^ on prokaryotic and microeukaryotic community composition in water and sediment samples from the same river. We expect distinct communities in the water and sediment for both prokaryotes and microeukaryotes. Furthermore, we anticipated more pronounced shifts in the composition of the pelagic community, as microorganisms in sediments typically inhabit a more stable environment, which may make them more resistant to abiotic disturbances (Han et al. 2023). Given the high prevalence of additive effects from multiple stressors observed in freshwater habitats (Velasco et al. 2018), we anticipate greater shifts in community composition for both bacteria and microeukaryotes when exposed to both stressors simultaneously, compared to exposure to a single stressor.

## 2 Materials and Methods

### 2.1 Experimental Setup and Stressor Experiment

To investigate the effects of increased temperature and salinisation on microbial stream communities, circular mesocosms (AquaFlow system) were used to conduct three identical experiments in September 2022, October 2022, and September 2023, using water and sediment from the Boye catchment, which is located in an urbanised region of North Rhine‐Westphalia, Germany (51°33′19.7″ N 6°56′38.3″ E). The Boye is a carbonate‐rich, sand‐dominated lowland stream with a history of sewage pollution but is currently undergoing ecological recovery as part of the restored Emscher river system (Perini 2017; Winking et al. 2014). The AquaFlow system was located in a greenhouse at the University of Duisburg–Essen and has been described in detail by Graupner et al. (2017). Sediment and water were transported to the experimental facility immediately after collection and transferred into the mesocosms on the same day to minimise abiotic and biotic changes prior to the experiment.

For each experiment, four identical mesocosms were constructed by connecting three steel tanks, with capacities of 40, 40, and 270 L, respectively, using two steel flow channels: one measuring 5 cm in width and 4 m in length, and the other 10 cm in width and 2 m in length, to replicate the riffle and pool sequences typical of natural streams. A pump (Eheim compactON 1000, Deizisau, Germany) and an aquarium chiller (TR/TC20, TECO SRL, Ravenna, Italy) were used to maintain water flow in each mesocosm and to control the water temperature. In a fifth mesocosm, hereafter referred to as the donor system, one of the 40 L tanks was replaced by a larger storage tank to allow for more water circulation in this system. The sediment retrieved from the stream was homogenised and mixed with fine particulate organic matter (FPOM), in a ratio of 9 parts sediment to 1 part FPOM, to enhance the nutrient content in the systems. Hereafter, the term ‘sediment’ will be used to refer to this mixture of sediment and FPOM. Forty‐five stainless steel mesh cylinders (height = 9 cm; diameter = 3 cm) were filled with sediment and placed evenly into each flow channel. The cores were then surrounded and covered by additional sediment to create a 10 cm high sediment layer in the channels. In total, 40 L of sediment were added into the two channels of each mesocosm. To simulate leaf litter, leaves of *Alnus glutinosa* were collected from trees in autumn, air‐dried, and cut into small pieces to be packed into mesh bags (3 × 5 cm^2^; 0.5 cm mesh, 0.6–0.8 g leaves/bag). Mixed species leaf litter from the Boye catchment was collected from the stream bed and incubated with the leaf bags before each experiment for 5 days to allow microbial communities native to the investigated stream to colonise the leaves. Afterwards, 35 leaf bags were attached on top of the cores in the narrow flow channel with stainless steel thread.

A 100 μm mesh was used to filter the water collected from the stream to remove debris and invertebrates, before 365 L of the filtered water was added to each mesocosm, except for the donor system, which was filled with approximately 1600 L of water. In the narrow channel, flow velocity was adjusted to 12 cm·s^−1^ and in the broad flow channel to 6 cm·s^−1^. Natural daylight was used as the light source throughout the entire experiment, and no artificial lighting or fixed light–dark cycles were applied. For 10 days, all mesocosms were acclimated while maintaining the water temperature at 15°C, mimicking typical local conditions in early autumn. During this period, daily water exchanges between the systems were performed to homogenise the microbial communities among the mesocosms. On the first day after the acclimatisation phase, hereafter referred to as Day 1, the abiotic conditions remained the same in all mesocosms, but water was no longer exchanged between the systems. On Day 2, the mesocosms were subjected to their respective stressors. In the temperature treatment and the combination treatment (temperature and salinity), water temperature was increased from 15°C to 20°C. Meanwhile, 2.5 g·L^−1^ of sodium chloride was added to both the salt treatment and the combination treatment. The abiotic conditions in the control and the donor system remained unchanged. To remove the stressors on day 6, three litres of water were collected from each treatment and divided into 250 mL dialysis flasks (Slide‐A‐LyzerTM dialysis flask, 20 K MWCO, 250 mL, Thermo ScientificTM, Waltham, Massachusetts, USA) before the remaining water in the control, temperature, salt, and combination treatments was fully drained. The four mesocosms were then refilled with water from the donor system and the dialysis flasks were placed into the 270 L tank of each corresponding mesocosm. The water temperature was maintained at 15°C until the end of the experiment on Day 10.

### 2.2 Sampling

Sediment was sampled daily by removing four cores from the narrow channel of each mesocosm. The resulting gaps in the sediment layer were filled with sterile sand to maintain the stability of the sediment layer. Sediment from the cores was homogenised and aliquoted into five 2 mL tubes, each containing 2 g of the homogenised sediment. The aliquots were frozen in liquid nitrogen and stored at −80°C until further processing for DNA extraction. Water samples (250 mL) were collected on days 1, 2, 3, 4, 5, 6, 8, and 10 and filtered onto white polycarbonate filters (diameter 47 mm, pore size 0.2 μm, Millipore GTTP 04700, Eschborn, Germany). The filters were air‐dried, frozen in liquid nitrogen, and stored at −80°C until further processing for DNA extraction. On Day 2, samples were collected 3 h after the stressors were added, and on Day 6, 3 h after stressor removal. This timing was chosen to capture the transitional phases shortly after stress onset and following its release. Abiotic conditions, including water temperature, pH, conductivity, salinity, and dissolved oxygen in both water and sediment, were measured daily. The corresponding measurements are provided in Table S1.

### 2.3 DNA Extraction and DNA Amplicon Sequencing

To prepare the samples for DNA extraction, 0.5 g of each sediment sample was aliquoted, and the water filters were carefully fragmented into small pieces using sterile tweezers. All samples were mixed with 2 mm Zirconia Beads (BioSpec Products, Bartlesville, OK, USA) and 0.3 mm Garnet Beads (BioSpec Products, Bartlesville, OK, USA). Then, 100 μL of Proteinase K (7bioscience GmbH, Neuenburg, Germany), 5 μL of RNase A (Qiagen, Hilden, Germany), and 900 μL of TNES (for buffer see Buchner 2022a) were added to both sediment and water samples, before bead‐beating them for 20 min at 2400 rpm using a Mini‐Bead‐Beater 96 (Biospec Products, Bartlesville, OK, USA), followed by a 20‐min incubation at 56°C and 1400 rpm. Two 300 μL aliquots of the resulting lysate (technical replicates) were processed for DNA extraction using the spin column method with a vacuum manifold as described in Buchner (2022b). The extracted DNA was purified with carboxylated magnetic beads and a PEG‐NaCl buffer as outlined by Buchner (2022c), using 40 μL of DNA and 80 μL of clean‐up solution for each sample. Via a two‐step PCR approach, using the Multiplex PCR Plus Kit (Qiagen, Hilden, Germany), the V4 region of the 16S rRNA genes and the V9 region of the 18S rRNA genes were amplified. For amplification, 1 μL of DNA and the primer pairs 515f (5′‐GTGCCAGCMGCCGCGGTAA‐3′)/806r (5′‐GGACTACHVGGGTWTCTAAT‐3′) for the 16S rRNA gene (Caporaso et al. 2011) and 1389F (5′‐TTGTACACACCGCCC‐3′)/1510R (5′‐CCTTCYGCAGGTTCACCTAC‐3′) for the 18S rRNA gene (Amaral‐Zettler et al. 2009) were used. The primer pairs were selected due to their broad coverage and frequent use in assessing bacterial and microeukaryotic communities, which ensures reliable and comparable results across studies. The PCR product was cleaned up following the protocol by Buchner (2022c) and 2 μL of the purified DNA was used for the second PCR, in which individual tags were added to each sample. PCR reagents and cycling conditions for both PCRs are listed in the Table S2. The PCR product of the second PCR was cleaned up using magnetic beads (Buchner 2022b) and then normalised prior to pooling. The resulting libraries were concentrated via the spin column clean‐up protocol by Buchner (2022b), before they were sent for paired‐end sequencing on Illumina NovaSeq (16S: 2 × 250 bp, CeGat, Tübingen, Germany; 18S: 2 × 150, Azenta Life Sciences, Leipzig Germany).

### 2.4 Bioinformatic Processing

The raw reads were processed with the Natrix2 amplicon workflow (v1.0.0, Deep et al. 2023), which included the main steps: primer removal, assembly using Pandaseq (v2.11, Masella et al. 2012), and read filtering based on an alignment threshold score of 0.9, with sequence lengths capped at a maximum of 600 bp and a minimum of 100 bp. Additional processing involved dereplication at 100% sequence similarity using cd‐hit (v4.8.1, Fu et al. 2012) and the removal of chimeric sequences via VSEARCH (v2.15.2, Rognes et al. 2016). Artificial sequences were removed via a split sample approach using AmpliconDuo (v1.1, Lange et al. 2015) before the remaining sequences were clustered into operational taxonomic units (OTUs) using the single‐linkage clustering method Swarm (v3.0.0, Mahé et al. 2022) with a default local clustering threshold of *d* = 1. For taxonomic classification, mothur (v1.47.0, Schloss et al. 2009) aligned the 16S rRNA gene sequences against the Silva database (v138.1, Quast et al. 2013) and the 18S rRNA gene sequences against the PR2 database (v4.14.0, Guillou et al. 2013), applying a minimum confidence value of 80, search = ‘kmer’, and classification method ‘wang’. Post‐clustering analysis was performed using MUMU (v0.0.1, Mahé 2022) to remove artificial OTUs. If not stated otherwise, all tools were used with default settings.

### 2.5 Data Analysis

All graphical and statistical analyses were carried out in RStudio (v4.3.1, PositTeam 2024) using the following packages: DESeq2 (v1.40.1, Love et al. 2014), dunn.test (v1.3.6, Dinno 2024), dplyr (v1.1.4, Wickham et al. 2023), effsize (Torchiano 2020), fantaxtic (v0.2.1, https://github.com/gmteunisse/fantaxtic), phyloseq (v.1.46.0, McMurdie and Holmes 2013), tidyverse (v2.0.0, Wickham et al. 2019) and vegan (v2.6.4, Oksanen et al. 2024). Visualisation of data was carried out using cowplot (v1.1.1, Wilke 2020), ggforce (v0.4.1, Pedersen 2022), ggpubr (v0.6.0, Kassambara 2023), ggplot2 (v3.5.1, Wickham 2016), ggh4x (v0.2.8, van den Brand 2024) and microViz (v0.12.1, Barnett et al. 2021).

Before downstream analysis, raw OTU tables for both 16S and 18S datasets were subjected to a series of quality filtering steps to remove likely sequencing artefacts and non‐target taxa. For both datasets, OTUs with missing taxonomic annotations (NA entries) or taxonomic labels assigned as unknown, unclassified entries, Archaea, Embryophyta, Metazoa, Chloroplasts, Mitochondria and non‐target domains (Eukaryota in the 16S dataset and Bacteria in the 18S dataset) were removed. To reduce noise from low‐abundance features, OTUs exclusive to one sample, as well as those with fewer than 100 reads, were removed before combining reads from both technical replicates. Additionally, the number of reads of OTUs identified in negative controls was subtracted from the corresponding counts in the samples. Read and OTU counts at different processing stages for 16S and 18S rRNA gene amplicon data in water and sediment samples are listed in Table S3. For each dataset, samples from the initial time point (Day 0, time point of experimental setup) and known outliers (day 6 of experiment 2 of water samples due to insufficient sequencing depth) were excluded.

For all analyses, data from the three identical experiments were combined, with each experiment considered as a biological replicate. The Gini‐Simpson index (1—D), hereafter referred to as Simpson's Diversity index, was calculated separately for 16S and 18S datasets using the estimate_richness function of the R package phyloseq. Resulting diversity values were merged with corresponding metadata for downstream analysis and visualisation. Due to the non‐normal distribution of alpha diversity data, as assessed by the Shapiro–Wilk test, we used non‐parametric Kruskal–Wallis tests to assess the influence of treatment during the stressor phase and sample source on alpha diversity. Alpha diversity was visualised as boxplots grouped by treatment and habitat showing variation across sampling days using ggpubr.

To assess the community composition over time and across treatments, taxonomic profiles were generated separately for the 16S and 18S datasets, using relative abundance data. First, the most abundant taxonomic groups were identified using the nested_top_taxa function from the fantaxtic package, retaining the top 10 taxa across all samples at phylum level and the three most abundant families nested within each of these phyla. All remaining families were grouped as ‘Other’.

To assess the overall structure of the microbial communities, beta diversity was analysed using Bray–Curtis dissimilarities. Prior to analysis, OTU count data were normalised using the DESeq2 package. Therefore, a DESeq2 object was created using the DESeqDataSetFromMatrix function (design formula: ~source + treatment). Size factors were estimated to account for library size differences, and normalised count values were obtained using the counts function. Finally, normalised values were log‐transformed using a natural log with pseudocount (log1p), before assessing general patterns via principal coordinates analysis (PCoA) based on Bray–Curtis dissimilarities. Ordination plots were generated separately for the 16S and 18S datasets with the ordinate and plot_ordination functions from the phyloseq package. Separate PCoA plots were generated to visualise microbial community differences across habitats (pelagic vs. benthic), temporal dynamics, and treatment effects. Points in the main figures are coloured or shaped to represent sampling times, treatments, or replicates as appropriate (Figures 2, 3, 4). Additional ordination plots detailing treatment and replicate effects are provided in the Supporting Information Figures. PCoA axes were selected based on the dimensions that best represented the variation of interest for each specific analysis.

Based on insights from the PCoA plots, separate partial distance‐based redundancy analyses (db‐RDAs) were performed using the capscale function from the vegan package to assess the independent effects of time (~time point, condition = ~treatment + replicate), replicate variation (~replicate, condition = ~treatment + time point), sample source (~source, condition = ~treatment + time point), and treatment (~treatment, condition = ~time point + replicate) on microbial community composition. This approach allowed us to isolate and test the unique contribution of timepoint, replicate, sample source and treatment, addressing confounding variation inherent in our experimental design. All partial db‐RDAs were based on Bray‐Curtis dissimilarities calculated from normalised (DESeq2, design formula: ~1, version 1.42.0) and log‐transformed (log1p) data. Time and replicate effects were evaluated over the entire experiment (Days 1–10), while treatment effects were assessed during the stressor phase (Days 2–5). The first db‐RDA axis scores were extracted and statistically compared. The significance of the time effect and replicate variation was tested using the Kruskal–Wallis test, due to the non‐normal distribution of the axis score data, and the effect size of the temporal effect was quantified using Eta‐squared (*η* ^2^), calculated from the Kruskal–Wallis test statistic. The significance of the treatment effect during the stressor phase was tested using the Kruskal–Wallis test, followed by Dunn's test for pairwise comparisons between treatments, implemented with the dunn.test function from the dunn.test package. Cliff's delta, a non‐parametric measure of effect size that quantifies the magnitude of differences between groups, was calculated using the cliff.delta function from the effsize package.

To compare microbial community compositions among treatments before stressor addition (Day 1) and at the end of the recovery phase (Day 10), nested PERMANOVAs were conducted on Bray–Curtis dissimilarity matrices, with replicates nested within treatment to account for variation among replicates.

To identify significant differences in the abundances of OTUs between the treatment systems and the control differential abundance analysis was performed separately for water and sediment samples across treatments and timepoints. For each combination of treatment, habitat (water or sediment), and time point, samples were subsetted accordingly. Count data were converted to DESeq2 objects, and differential abundance testing was performed using the DESeq2 pipeline with the model ~ treatment. Results comparing each treatment against control were extracted and highly significant OTUs were identified based on an adjusted *p* value threshold of 0.01.

## 3 Results

### 3.1 Varying Temporal Effect on Distinct Communities in Water and Sediment

**FIGURE 1:**
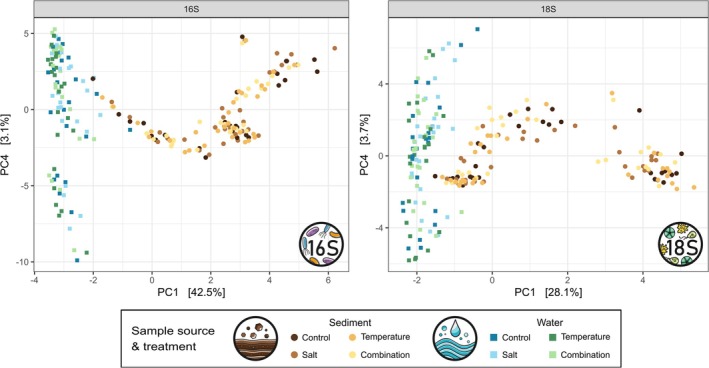
Distinct microbial communities in water and sediment samples. Visualisation via principal coordinates analysis (PCoA) based on Bray–Curtis dissimilarity matrices for prokaryotic (left) and microeukaryotic (right) communities. The PCoA plot illustrates the separation between pelagic and benthic microbial communities along axis 1.

Principal coordinates analysis (PCoA) plots revealed distinct community compositions in water and sediment samples (Figure 1). This visual separation was supported by partial distance‐based redundancy analysis (db‐RDA) when testing for the effect of sample source on microbial community composition while controlling for treatment and time effects. Significant differences in OTU composition between sources were observed for both prokaryotes and microeukaryotes (Kruskal–Wallis Test on db‐RDA scores, *p* < 0.001 for both groups).

**FIGURE 2:**
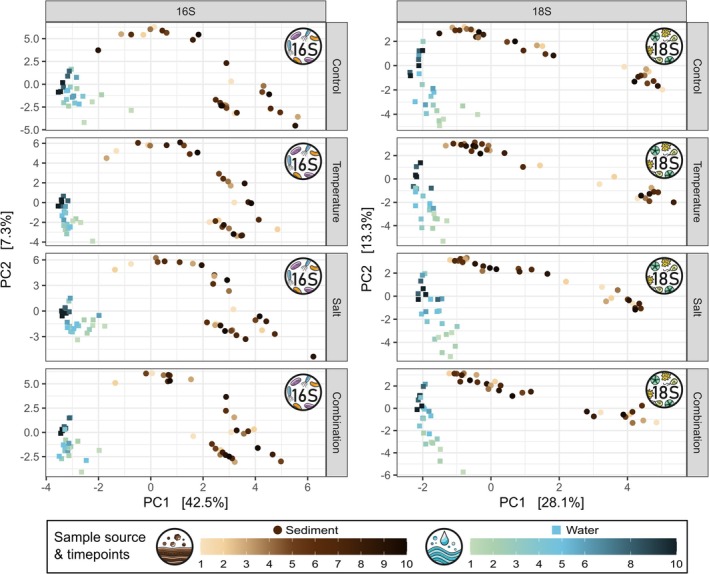
Varying temporal effect on prokaryotic and microeukaryotic communities in water and sediment samples. Visualisation via PCoA based on Bray–Curtis dissimilarity matrices for prokaryotic (left) and microeukaryotic communities (right) over time with points coloured by time point and facetted by treatment. The PCA plots reveal a strong temporal effect on axis 2 in the pelagic (blue squares) but not in the benthic community (brown circles).

Additionally, a temporal effect on microbial community composition was observed in both water and sediment habitats (Figure 2). Partial db‐RDA analyses, controlling for treatment and replicate variation, revealed significant impacts of sampling time point on prokaryotic communities (Kruskal–Wallis Test on db‐RDA scores: water *p* < 0.001, sediment *p* = 0.001) and microeukaryotic communities (water *p* < 0.001, sediment *p* = 0.01) across all treatments. Interestingly, the effect size of this temporal effect varied strongly between the habitats, being strongest in water, where it explained 39.2% of the variance in the prokaryotic and 40.9% in the microeukaryotic communities. In contrast, the temporal effect in sediment was lower, accounting for only 18.6% and 15.2% of the variance in the prokaryotic and microeukaryotic communities, respectively. Due to the temporal separation of the experiments, the initial microbial communities differed significantly among experiments (Figure S1). This was supported by partial db‐RDA analyses controlling for treatment and timepoint, revealing significant differences among the three biological replicates for both prokaryotes (Kruskal–Wallis Test on db‐RDA scores: water *p* < 0.001, sediment *p* < 0.001) and microeukaryotes (water *p* < 0.001, sediment *p* < 0.001).

### 3.2 Varying Responses of the Microbial Community to Salinisation in Water and Sediment

Before the addition of stressors (day 1), no significant differences were observed between the prokaryotic communities in the control and treatment mesocosms in both water (nested PERMANOVA controlling for replicate variation, temperature: *p* = 0.837; salt: *p* = 0.309; combination: *p* = 0.657) and sediment (nested PERMANOVA controlling for replicate variation, temperature: *p* = 0.341; salt: *p* = 0.577; combination: *p* = 0.380). Similarly, no significant differences were detected between the microeukaryotic communities in the control and treatment mesocosms in the water (nested PERMANOVA controlling for replicate variation, temperature: *p* = 0.158; salt: *p* = 0.750; combination: *p* = 0.237) or in the sediment (nested PERMANOVA controlling for replicate variation, temperature: *p* = 0.529; salt: *p* = 0.937; combination: *p* = 0.295).

**FIGURE 3:**
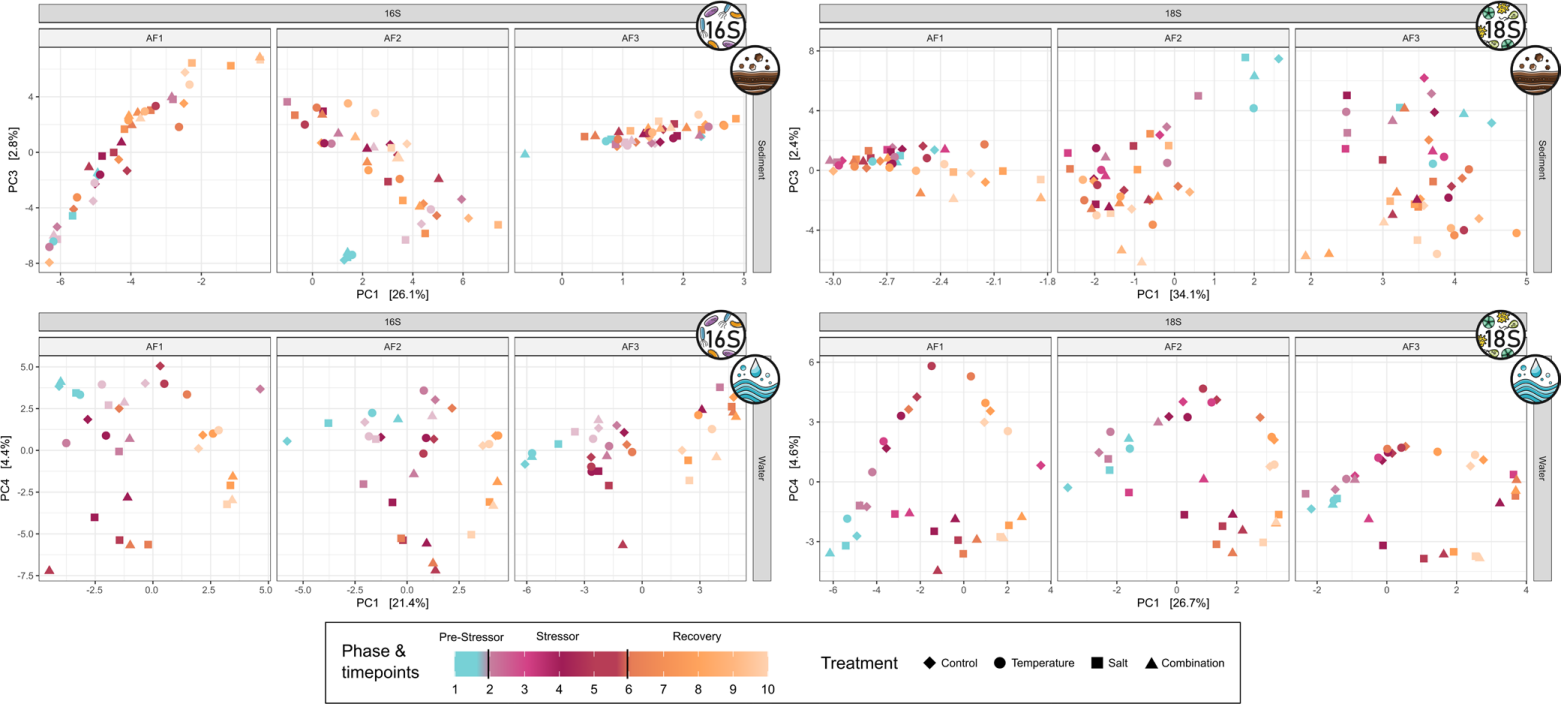
Treatment effect on prokaryotic and microeukaryotic communities in water and sediment samples. Visualisation via PCoA plots based on Bray–Curtis dissimilarities calculated from the combined data of all three replicate experiments, showing prokaryotic (left) and microeukaryotic (right) communities in sediment (top) and water (bottom) samples. Each plot displays community changes over time, with treatment groups represented by different colours and shapes, and facetted by replicate to better visualise treatment effects.

PCoA plots combining data from all three experiments revealed that samples from the salt and combination treatments clustered together, while samples from the temperature treatment grouped with the controls (Figure S2). To better visualise treatment effects across experiments, separate PCoA plots are presented for each experiment, based on a single ordination calculated from the entire dataset (Figure 3). The patterns observed in these visualisations were supported by partial db‐RDAs testing for treatment effects while controlling for time point and replicate variation. These analyses detected significant differences among treatments in the pelagic microbial communities for both prokaryotes and microeukaryotes (Kruskal–Wallis Test on db‐RDA scores, *p* < 0.001 for both groups). In contrast, no significant treatment effects were found in the benthic communities (prokaryotes: *p* = 0.212; microeukaryotes: *p* = 0.066).

Differences among treatments were assessed using Dunn's Test on the db‐RDA scores. The pelagic community in the temperature treatment did not significantly differ from that in the control for both prokaryotes (*p* = 0.397) and microeukaryotes (*p* = 0.137). The increase in salinity, however, significantly altered the community composition for both prokaryotes and microeukaryotes, resulting in significant differences between the microbial communities in the control and the salt treatment (prokaryotes: *p* < 0.001; microeukaryotes: *p* < 0.001) as well as between the control and the combination treatment (prokaryotes: *p* = 0.021; microeukaryotes: *p* < 0.001). Similarly, the temperature treatment significantly differed from both salt (prokaryotes: *p* < 0.001; microeukaryotes: *p* < 0.001) and combination treatment (prokaryotes: *p* = 0.011; microeukaryotes: *p* < 0.001). Significant differences between the salt and the combination treatment were observed for the prokaryotic (*p* = 0.043) but not for the microeukaryotic community (*p* = 0.391).

Cliff's delta was calculated to assess the magnitude of differences between treatments (Table 1). The salt treatment had the strongest effect on microbial community composition. For prokaryotes, the effect size of the combination treatment was smaller than that of the salt treatment alone, whereas for microeukaryotes, the effect sizes of the salt and combination treatments were much more similar. Overall, effect sizes were higher for the microeukaryotes than for the prokaryotes.

**TABLE 1:** Ranked effect sizes (Cliff's Delta) for pairwise treatment comparisons showing the impact on prokaryotic and micro‐eukaryotic communities in the water column during the stressor phase.

Rank	Comparison	Effect size	Absolute effect size
Prokaryotic community			
1	Salt—temperature	0.847	0.847
2	Control—salt	−0.819	0.819
3	Combination—temperature	0.667	0.667
4	Salt—combination	0.597	0.597
5	Control—combination	−0.555	0.555
6	Control—temperature	0.056	0.056
Microeukaryotic community			
1	Salt—temperature	−0.972	0.972
2	Combination—temperature	−0.944	0.944
3	Control—salt	0.903	0.903
4	Control—combination	0.861	0.861
5	Control—temperature	−0.444	0.444
6	Salt—combination	−0.097	0.097

Despite the distinct starting communities, similar treatment effects were observed in all three replicates when each experiment was analysed individually (Figure S3). The results of significance tests for each individual experiment are listed in Table S4.

### 3.3 Communities in Salt and Combination Treatments Remain Significantly Different After Stressor Removal

At the end of the recovery phase (Day 10), significant differences were observed in the pelagic microbial communities between the control and the salt treatment (nested PERMANOVA controlling for replicate variation, prokaryotes: *p* = 0.01; microeukaryotes: *p* = 0.01). Similarly, significant differences between the control and combination treatment were observed for both prokaryotes (nested PERMANOVA controlling for replicate variation, *p* = 0.003) and microeukaryotes (nested PERMANOVA controlling for replicate variation, *p* = 0.004). No significant differences were observed between the control and the temperature treatment (nested PERMANOVA controlling for replicate variation, prokaryotes: *p* = 0.475; microeukaryotes: *p* = 0.769).

In the sediment, no significant differences between the control and any of the treatments were observed in the prokaryotic community (nested PERMANOVA controlling for replicate variation, temperature: *p* = 0.290; salt: *p* = 0.071; combination: *p* = 0.119). The microeukaryotic community significantly differed between the control and the combination treatment (nested PERMANOVA controlling for replicate variation, *p* = 0.035) but not between the control and the single stressor treatments (nested PERMANOVA controlling for replicate variation, temperature: *p* = 0.101; salt: *p* = 0.153).

### 3.4 Alpha‐Diversity of Microbial Communities in Water and Sediment

**FIGURE 4:**
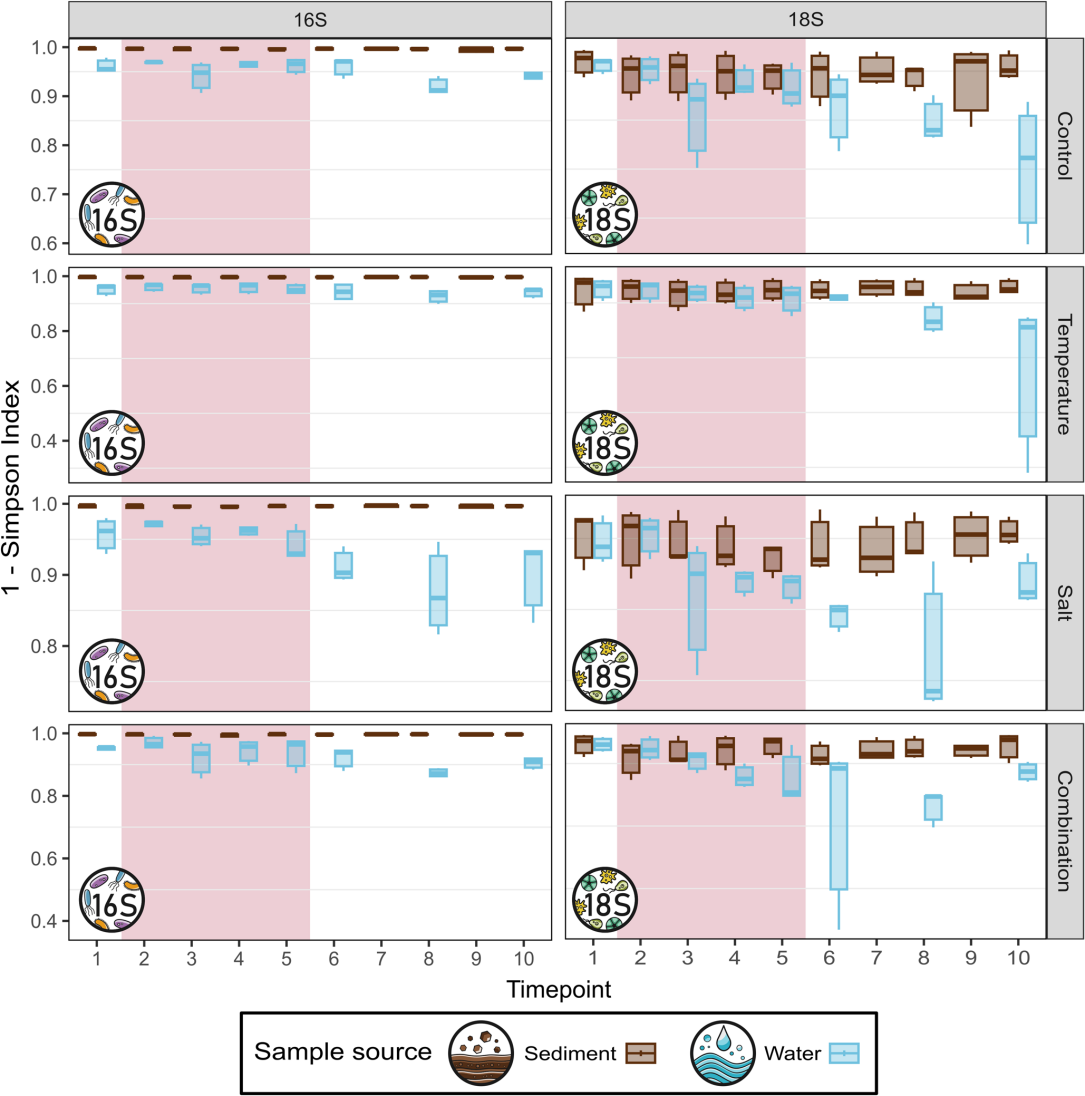
Alpha‐diversity of microbial communities in water and sediment. Simpson's diversity indices of the prokaryotic (left) and microeukaryotic communities (right) in each treatment throughout the experiments in sediment (brown) and water samples (blue). Days of stressor exposure are highlighted in red.

The Simpsons' Diversity indices significantly differed between communities in the water and in the sediment for both prokaryotes (Kruskal–Wallis Test, *p* < 0.001) and microeukaryotes (Kruskal–Wallis Test, *p* < 0.001). The diversity of the microbial communities in the water was generally lower than in the sediment (Figure 4). No significant differences were observed among treatments for the prokaryotic community in the water (Kruskal–Wallis Test, *p* = 0.567) or in the sediment (Kruskal–Wallis Test, *p* = 0.954). Similarly, no significant differences were observed among treatments for the microeukaryotic community in the water (Kruskal–Wallis Test, *p* = 0.542) or in the sediment (Kruskal–Wallis Test, *p* = 0.905).

### 3.5 Taxonomic Composition of the Microbial Communities in Sediment and Water

In the sediment, microbial communities remained relatively stable throughout the experiment, with little variation across treatments. The prokaryotic community was dominated by Pseudomonadota—particularly Alpha‐ and Gammaproteobacteria—along with Bacteroidota, Desulfobacterota, Actinomycetota, and Acidobacteriota (Figure S4), while the microeukaryotic community was characterised by high abundances of Ochrophyta, specifically rapid pennate diatoms, as well as Streptophyta, Chlorophyta, Fungi, and unclassified eukaryotes (Figure S5). This compositional stability was reflected in the differential abundance analysis, which identified only a few OTUs with significantly different abundances in the sediment. However, these differences were confined to individual days and did not follow a consistent temporal pattern. In contrast, pelagic microbial communities were more dynamic and responsive to environmental changes. Although Pseudomonadota and Bacteroidota consistently dominated the prokaryotic communities across timepoints and treatments, compositional changes were evident in response to the salt and combination treatments, where the relative abundance of Actinomycetota, particularly Sporichthyaceae, was reduced, compensated mainly by increases in the relative abundance of Pseudomonadota and Flavobacteriaceae (Figure S4). Differential abundance analyses confirmed these patterns, with significantly enriched OTUs affiliated with Alphaproteobacteria and Flavobacteriaceae, and reduced abundance of OTUs assigned to Betaproteobacteria and Actinomycetota (Figure S6). The pelagic microeukaryotic community, initially dominated by unclassified eukaryotes, Chrysophyceae, and other Ochrophyta, also shifted in response to the salt and combination treatments (Figure S5). During the stressor phase (Day 2–5), unclassified Ochrophyta increased in the control and temperature treatments, while Chrysophyceae became more abundant in the salt and combination treatments. Interestingly, Chrysophyceae exhibited the highest relative abundance across all treatments by the end of the experiment (Figure S5). These dynamics were supported by the differential abundance analysis, which revealed a significant increase in OTUs classified as Chrysophyceae and a concurrent decline in OTUs assigned to other unclassified Ochrophyta in the salt and combination treatments (Figure S7). In contrast, the temperature treatment induced only minor changes in both the prokaryotic (Figure S6) and microeukaryotic communities (Figure S7) in the water, with relatively few OTUs differing significantly from the control. Similar to the benthic communities, significant differences in OTU abundance in pelagic microbial communities under the temperature treatment appeared on individual days and showed no consistent pattern over time.

## 4 Discussion

Microorganisms are integral to freshwater ecosystems (Falkowski et al. 2008; Linz et al. 2018), and environmental changes can significantly alter microbial communities, leading to shifts that can disrupt ecosystem functioning and stability (Beattie et al. 2020; Martin et al. 2020; Mo et al. 2021). Therefore, gaining insight into how both prokaryotic and microeukaryotic communities respond to environmental changes is crucial for understanding and predicting the effects of climate change and anthropogenic activities on freshwater habitats.

Our results show that water and sediment harbour distinct microbial communities (Figure 1), which is in accordance with previous studies that demonstrated that pelagic and benthic microbial communities in freshwater habitats significantly differ from each other (Lu et al. 2016; Li et al. 2023; Wu, Zhao, et al. 2023). The prokaryotic community in our experiment was representative of typical riverine water and sediment communities (Figure S4), with Pseudomonadota and Bacteroidota being the most abundant phyla in both habitats (Zwart et al. 2002; Tamames et al. 2010). Our results suggest that salinisation negatively impacted Actinomycetota while having a positive effect on Alphaproteobacteria (Figure S4), which aligns with previous studies (Gagnon et al. 2022; Lew et al. 2022). Alphaproteobacteria often exhibit higher abundances in environments with elevated salinity, suggesting greater tolerance to salinity stress, whereas Actinobacteria tend to decline as salinity increases (Chen et al. 2022; Lew et al. 2022). The pattern that Chrysophyceae were highly abundant in the water column and Bacillariophyta dominated the microeukaryotic community in the sediment is consistent with prior studies (Figure S5), as most Chrysophyceae tend to be planktonic (Sheath and Wehr 2015), while Bacillariophyta are typically highly abundant in benthic communities (Tsoi et al. 2017; Wu, Dong, et al. 2023). The relatively high abundance of Chrysophyceae in the salt and combination treatments also corresponds with findings by Astorg et al. (2022), who demonstrated their ability to withstand elevated chloride concentrations. Such shifts in community composition may impact important ecosystem functions, such as nutrient cycling and microbial predator–prey interactions. However, the ecological implications require further investigation. Interestingly, Chlorophyta and Streptophyta exhibited higher relative abundance in the sediment compared to the pelagic community (Figure S5), despite being commonly associated with phytoplankton (Korneva 2012; Belous et al. 2014; Jana 2024). This may be attributed to the low water level in the experimental setup, as benthic algae are known to dominate in shallow rivers (Jäger et al. 2017; Yang et al. 2021).

In our experiment, microbial communities in both water and sediment were influenced by a temporal effect, which is typical for such mesocosm experiments (Graupner et al. 2017; Sieber et al. 2023; Stach et al. 2023) and likely driven by the experimental setup selecting for a specific community (Figure 2). For example, the eventual dominance of Chrysophyceae in the microeukaryotic community across all treatments by the end of the experiment suggests that this group might benefit from conditions inherent to the experimental setup. However, this temporal effect was less pronounced in the sediment than in the water, suggesting greater stability of the benthic communities. Additionally, sediment communities proved to be more resistant to environmental changes. Increases in temperature and salinity, both individually and combined, had no significant effect on the composition of microbial communities in the sediment, while salinisation significantly altered the prokaryotic and microeukaryotic community in the water column (Figure 3). Previous studies demonstrated varying levels of resilience of benthic microbial communities to disturbances such as changes in land use and salt discharge (Martin et al. 2020; Tammert et al. 2023) and provided evidence that microbial communities in the sediment tend to be more resilient to environmental changes than pelagic communities (Tammert et al. 2023; Liao et al. 2019; Chang et al. 2020; Cai et al. 2020). The greater stability and resilience exhibited by benthic communities compared to pelagic communities are likely due to complex microhabitat structures that promote higher diversity in the sediment (Zhang et al. 2020; Li et al. 2023), which in turn increases resistance to environmental changes (Yachi and Loreau 1999; Guo et al. 2024). In our experiment, alpha‐diversity of the benthic communities was significantly higher than that of the pelagic communities (Figure 4), which may have enhanced resistance to increased salinity in the sediment. Furthermore, the more dynamic conditions of the water column can lead to rapid fluctuations in community composition, making them more susceptible to environmental changes (Hassell et al. 2018; Ren et al. 2019).

Although alpha diversity metrics remained largely stable across treatments for both benthic and pelagic communities, beta diversity analyses revealed significant compositional shifts in the pelagic community, suggesting that environmental stressors altered community structure without affecting overall richness or evenness. In the water column, the temperature treatment did not significantly affect the composition of either the prokaryotic or microeukaryotic community, despite previous research showing that temperature increases can significantly alter microbial community composition (Hao et al. 2018; Rocca et al. 2022; Engloner et al. 2023). Other than our study, which examined short‐term temperature increases, these studies focused on prolonged warming or seasonal temperature variations, indicating that microbial communities might exhibit greater resistance to brief periods of heat stress. This is particularly true given the time of sample collection from the river, a period that is typically marked by cooling temperatures but occasional heat spikes, indicating that microbes had probably not fully adjusted to autumn conditions at the time.

In contrast to the temperature treatment, salinization significantly altered the pelagic microbial communities in our experiments (Figure 3), with a more pronounced effect on microeukaryotes than on prokaryotes (Table 1). This could be explained by the higher cellular complexity of microeukaryotes, which rely on intricate osmoregulatory mechanisms. Prokaryotes, on the other hand, employ a broader range of strategies to cope with osmotic stress (Gunde‐Cimerman et al. 2018). Given their heightened sensitivity to elevated salt levels, microeukaryotes are considered more suitable for assessing salinization effects in freshwater environments (Gagnon et al. 2022). Nonetheless, the sensitivity of prokaryotic and microeukaryotic communities can vary depending on the type of stressor, as other studies have suggested that bacteria may be more sensitive to environmental changes (Pearman et al. 2020; Fang et al. 2021; Lu et al. 2022). This highlights the importance of considering both prokaryotic and microeukaryotic communities when monitoring the impact of environmental changes in natural habitats.

Interestingly, prokaryotic communities in the combination treatment significantly differed from the community in the salt treatment, while there were no significant differences between salt and combination treatment in the microeukaryotic community. While we did not observe any significant effects of the temperature treatment alone, increased temperature may have enhanced the salt tolerance of prokaryotes, thereby reducing the impact of increased salinity. The prokaryotes may not have experienced the elevated water temperature of 20°C as a stressor, as it may have been closer to the optimum temperature for many species (Kritzberg and Bååth 2022). In contrast, susceptibility to salinisation of microeukaryotes may be less affected by changes in water temperature (Fang et al. 2021; Thakur et al. 2021), which could explain the similar effects observed in microeukaryotic communities exposed to salt and combination treatment.

During the recovery phase, pelagic prokaryotic and microeukaryotic communities in the salt and combination treatment remained significantly different from the control, suggesting that the communities had not recovered by the end of the experiment. Additionally, during the recovery, significant differences emerged between the benthic microeukaryotic community in the control and the combination treatment, while no such differences were observed during the stressor phase, suggesting delayed stressor effects. Consequently, longer stressor and recovery periods are necessary to fully evaluate the impact of increased temperature and salinity on both pelagic and benthic communities and to determine if complete recovery is possible following such disturbances.

The treatment effects were consistent across all three experiments despite distinct starting communities (Figure S3), indicating that their impact is independent of the initial composition of the microbial communities. This consistency suggests that the observed patterns are not specific to a particular community and can be generalised beyond the limits of a distinct starting community (Beentjes et al. 2019).

## 5 Conclusion

Our findings demonstrated the varying responses of riverine microbial communities to environmental changes. Pelagic communities were more affected by salinisation than benthic communities, likely due to the greater stability and diversity of microbial communities in sediments. Furthermore, microeukaryotes exhibited stronger responses to salinisation than prokaryotes, suggesting their potential as valuable bioindicators when monitoring the impact of environmental disturbances. By incorporating diverse starting communities, our experiments demonstrated that these effects are not limited to specific community compositions, which allows for a broader generalisation of our results.

## Author Contributions

**Lisa Boden:** formal analysis, investigation, resources, validation, visualisation, writing – original draft, writing – review and editing. **Dana Bludau:** formal analysis, methodology, software, validation, visualisation, writing – review and editing. **Guido Sieber:** conceptualisation, investigation, project administration, resources, writing – review and editing. **Aman Deep:** data curation, investigation, methodology, software, validation, writing – review and editing. **Daria Baikova:** investigation, writing – review and editing. **Gwendoline M. David:** investigation, writing – review and editing. **Una Hadžiomerović:** investigation, writing – review and editing. **Tom L. Stach:** investigation, writing – review and editing. **Dominik Buchner:** data curation, investigation, methodology, resources, validation, writing – review and editing. **Jens Boenigk:** conceptualisation, funding acquisition, project administration, supervision, writing – review and editing.

## Conflicts of Interest

The authors declare no conflicts of interest.

## Supporting information


**Table S1:** Daily abiotic measurements for each mesocosm on each sampling day. Values represent the average of three replicate measurements. Units for each parameter are indicated in the table headings.
**Table S2:** Reagents and cycling conditions of the first and the second PCR for 16S and 18S rRNA gene amplification. 20 cycles were used for the first PCR and 25 cycles were used for the second PCR.
**Table S3:** Summary of read and OTU Counts at different processing stages for 16S and 18S data in water and sediment samples.
**Figure S1:** Temporal effect and replicate variation of benthic and pelagic microbial communities. Visualisation via PCoA based on Bray–Curtis dissimilarity matrices for prokaryotic (left) and microeukaryotic community (right) in water (top) and sediment (bottom). Replicates are represented by different shapes, while the different sampling days are represented by a colour gradient.
**Figure S2:** Treatment effects of benthic and pelagic microbial communities. Visualisation via PCoA based on Bray–Curtis dissimilarity matrices for prokaryotic (left) and eukaryotic microbial (right) community in water (top) and sediment (bottom). The different treatments are visualised by different colours and shapes.
**Figure S3:** Treatment effect on prokaryotic and microeukaryotic communities in water and sediment in each individual experiment. Visualisation based via PCoA and Bray–Curtis dissimilarity matrices for prokaryotic (left) and microeukaryotic community (right) in sediment (top) and water samples (bottom) from the individual experiments. PCoA plots depict treatment effect on axes 1 and 2 for AquaFlow experiment 1 (AF1; conducted in September 2022), AquaFlow experiment 2 (AF2; conducted in September 2022) and AquaFlow experiment 3 (AF3; conducted in September 2023). The data for each experiment was analysed separately.
**Table S4:** emi70173‐sup‐0001‐supinfo.docx. *p* values from nested PERMANOVA testing the effect of treatments on microbial community composition while accounting for temporal variation for each individual experiment during the stressor phase. The data for each experiment was analysed separately.
**Figure S4:** Taxonomic composition of the prokaryotic communities in water (top) and sediment (bottom) exposed to different treatments. Bar plots display the ten most abundant phyla and their three most abundant families. Families within the same phylum are represented by similar colours. ‘Other’ encompasses all additional taxa present in the samples. Samples were collected after acclimation but before stressor addition (D01), during the stressor phase (D02‐D05), and after stressor removal (D06‐D10).
**Figure S5:** Taxonomic composition of the microeukaryotic communities in water (top) and sediment (bottom) exposed to different treatments. Bar plots display the ten most abundant phyla and their three most abundant families. Families within the same phylum are represented by similar colours. ‘Other’ encompasses all additional taxa present in the samples. Samples were collected after acclimation but before stressor addition (D01), during the stressor phase (D02‐D05), and after stressor removal (D06‐D10).
**Figure S6:** Differentially abundant OTUs across treatments and time in the prokaryotic community. Heatmaps visualising temporal dynamics of the top 50 differentially abundant OTUs (ranked by maximum absolute log2 fold change) per treatment and sample source across time points. Colour intensity represents log2 fold change relative to the control. OTU labels include taxonomic information at the family level.
**Figure S7:** Differentially abundant OTUs across treatments and time in the microeukaryotic community. Heatmaps visualising temporal dynamics of the top 50 differentially abundant OTUs (ranked by maximum absolute log2 fold change) per treatment and sample source across time points. Colour intensity represents log2 fold change relative to the control. OTU labels include taxonomic information at the family level.

## Data Availability

Amplicon sequence data generated and analysed in this study has been deposited in the NCBI SRA repository under accession number PRJNA1178677).
