# Supplementary material for: Varying Responses to Heat Stress and Salinization Between Benthic and Pelagic Riverine Microbial Communities
Source: Environ Microbiol. 2025 Sep 4;27(9):e70173. doi: 10.1111/1462-2920.70173 (PMC12411665; doi:10.1111/1462-2920.70173)
Supplement: Supplementary file 1 — Table S1: Daily abiotic measurements for each mesocosm on each sampling day. Values represent the average of three replicate measurements. Units for each parameter are indicated in the table headings. Table S2: Reagents and cycling conditions of the first and the second PCR for 16S and 18S rRNA gene amplification. 20 cycles were used for the first PCR and 25 cycles were used for the second PCR. Table S3: Summary of read and OTU Counts at different processing stages for 16S and 18S data in water and sediment samples. Figure S1: Temporal effect and replicate variation of benthic and pelagic microbial communities. Visualisation via PCoA based on Bray–Curtis dissimilarity matrices for prokaryotic (left) and microeukaryotic community (right) in water (top) and sediment (bottom). Replicates are represented by different shapes, while the different sampling days are represented by a colour gradient. Figure S2: Treatment effects of benthic and pelagic microbial communities. Visualisation via PCoA based on Bray–Curtis dissimilarity matrices for prokaryotic (left) and eukaryotic microbial (right) community in water (top) and sediment (bottom). The different treatments are visualised by different colours and shapes. Figure S3: Treatment effect on prokaryotic and microeukaryotic communities in water and sediment in each individual experiment. Visualisation based via PCoA and Bray–Curtis dissimilarity matrices for prokaryotic (left) and microeukaryotic community (right) in sediment (top) and water samples (bottom) from the individual experiments. PCoA plots depict treatment effect on axes 1 and 2 for AquaFlow experiment 1 (AF1; conducted in September 2022), AquaFlow experiment 2 (AF2; conducted in September 2022) and AquaFlow experiment 3 (AF3; conducted in September 2023). The data for each experiment was analysed separately. Table S4: emi70173‐sup‐0001‐supinfo.docx. p values from nested PERMANOVA testing the effect of treatments on microbial community composition while accounti [file EMI-27-e70173-s001.docx]

**Supplementary information for**

**Varying responses to heat stress and salinization between benthic and pelagic riverine microbial communities**

Lisa Boden^1^, Dana Bludau^1,2,3^, Guido Sieber^1,2^, Aman Deep^1,3^, Daria Baikova^4^, Gwendoline David^5^, Una Hadžiomerović^2,4^, Tom L. Stach^2,6^, Dominik Buchner^7^ & Jens Boenigk^1,2^

*^1^ Department Biodiversity, University of Duisburg–Essen, Essen, Germany*

*^2^ Center for Water and Environmental Research, University of Duisburg–Essen, Essen, Germany*

*^3^ Department of Engineering and Natural Sciences, Westphalian University of Applied Sciences,*

*Recklinghausen, Germany*

*^4^ Department Environmental Microbiology and Biotechnology, University of Duisburg–Essen, Essen, Germany*

*^5^ Department of Plankton and Microbial Ecology, Leibniz Institute of Freshwater Ecology and Inland Fisheries (IGB), Stechlin, Germany*

*^6^ Environmental Metagenomics Research Center One Health Ruhr, University Alliance Ruhr, University of Duisburg–Essen, Essen, Germany*

*^7^ Department Aquatic Ecosystem Research, University of Duisburg-Essen, Essen, Germany*

**Table S1**. Daily abiotic measurements for each mesocosm on each sampling day. Values represent the average of three replicate measurements. Units for each parameter are indicated in the table headings.

| **Experiment 1 (September 2022)** | | | | | | | | |
| --- | --- | --- | --- | --- | --- | --- | --- | --- |
| **Day** | **Treatment** | | **O_2_ Sediment in mg/l** | **O_2_ Water in mg/l** | **Salinity in %** | **Conductivity in mS/cm** | **pH** | **Temperature in °C** |
| 1 | | Control | 3,783 ± 0,962 | 9,5 ± 0,1 | 0,03 ± 0 | 0,603 ± 0,001 | 8,523 ± 0,015 | 15,6 ± 0 |
| 1 | | Temperature | 4,117 ± 1,376 | 9,4 ± 0,1 | 0,03 ± 0 | 0,573 ± 0,004 | 8,513 ± 0,029 | 15,867 ± 0,058 |
| 1 | | Salt | 3,157 ± 0,576 | 9,3 ± 0 | 0,03 ± 0 | 0,595 ± 0,001 | 8,567 ± 0,006 | 15,6 ± 0 |
| 1 | | Combination | 4,43 ± 0,763 | 9,5 ± 0,1 | 0,03 ± 0 | 0,571 ± 0,002 | 8,647 ± 0,029 | 15,3 ± 0 |
| 2 | | Control | 5,407 ± 1,303 | 10,333 ± 0,569 | 0,03 ± 0 | 0,575 ± 0,001 | 8,627 ± 0,031 | 14,7 ± 0 |
| 2 | | Temperature | 5,797 ± 1,53 | 9,767 ± 0,85 | 0,03 ± 0 | 0,568 ± 0,004 | 8,423 ± 0,057 | 18 ± 0 |
| 2 | | Salt | 5,85 ± 1,413 | 9,933 ± 1,012 | 0,24 ± 0 | 4,517 ± 0,012 | 8,403 ± 0,006 | 15,567 ± 0,058 |
| 2 | | Combination | 5,68 ± 0,886 | 8,7 ± 0,173 | 0,22 ± 0 | 4,02 ± 0,01 | 8,513 ± 0,091 | 17,167 ± 0,058 |
| 3 | | Control | 6,653 ± 2,64 | 10,333 ± 0,569 | 0,03 ± 0 | 0,57 ± 0,001 | 8,703 ± 0,101 | 14,7 ± 0 |
| 3 | | Temperature | 6,05 ± 1,338 | 9,767 ± 0,85 | 0,03 ± 0 | 0,581 ± 0 | 8,447 ± 0,015 | 20,6 ± 0 |
| 3 | | Salt | 4,95 ± 1,335 | 9,933 ± 1,012 | 0,26 ± 0 | 4,967 ± 0,006 | 8,31 ± 0,026 | 15,6 ± 0 |
| 3 | | Combination | 6,057 ± 1,508 | 8,7 ± 0,173 | 0,26 ± 0 | 4,777 ± 0,029 | 8,443 ± 0,179 | 20,7 ± 0,1 |
| 4 | | Control | 3,947 ± 0,579 | 9,433 ± 0,231 | 0,03 ± 0 | 0,558 ± 0,005 | 8,727 ± 0,067 | 15,567 ± 0,208 |
| 4 | | Temperature | 3,243 ± 1,478 | 8,067 ± 0,493 | 0,03 ± 0 | 0,571 ± 0,001 | 8,737 ± 0,025 | 20,433 ± 0,058 |
| 4 | | Salt | 4,583 ± 2,137 | 9,867 ± 0,115 | 0,26 ± 0 | 4,98 ± 0,04 | 8,27 ± 0,062 | 15,033 ± 0,058 |
| 4 | | Combination | 3,7 ± 1,377 | 7,933 ± 0,153 | 0,26 ± 0 | 4,993 ± 0,025 | 8,563 ± 0,067 | 20,1 ± 0,1 |
| 5 | | Control | 5,553 ± 1,079 | 8,933 ± 0,289 | 0,03 ± 0 | 0,538 ± 0,014 | 8,293 ± 0,006 | 15 ± 0,1 |
| 5 | | Temperature | 5,2 ± 1,921 | 8,367 ± 0,058 | 0,03 ± 0 | 0,561 ± 0,001 | 8,27 ± 0,072 | 20,567 ± 0,058 |
| 5 | | Salt | 4,617 ± 2,547 | 9,2 ± 0,1 | 0,28 ± 0 | 5,057 ± 0,042 | 8,283 ± 0,006 | 16,2 ± 0,173 |
| 5 | | Combination | 4,127 ± 0,652 | 8 ± 0,265 | 0,273 ± 0,006 | 5,057 ± 0,015 | 8,343 ± 0,012 | 20,767 ± 0,058 |
| 6 | | Control | 4,96 ± 1,612 | 8,6 ± 0,361 | 0,03 ± 0 | 0,529 ± 0,001 | 8,643 ± 0,067 | 15,833 ± 0,058 |
| 6 | | Temperature | 4,52 ± 1,812 | 8,633 ± 0,306 | 0,03 ± 0 | 0,528 ± 0,001 | 8,577 ± 0,031 | 15,65 ± 0,071 |
| 6 | | Salt | 6,813 ± 2,005 | 8,633 ± 0,153 | 0,04 ± 0 | 0,721 ± 0,001 | 8,67 ± 0,01 | 14,467 ± 0,058 |
| 6 | | Combination | 5,58 ± 0,298 | 8,7 ± 0,361 | 0,04 ± 0 | 0,708 ± 0,001 | 8,63 ± 0,036 | 14,7 ± 0 |
| 7 | | Control | 2,467 ± 1,64 | 9,333 ± 0,058 | 0,03 ± 0 | 0,539 ± 0,002 | 8,557 ± 0,575 | 14,833 ± 0,058 |
| 7 | | Temperature | 5,883 ± 0,332 | 9,167 ± 0,058 | 0,03 ± 0 | 0,533 ± 0,001 | 8,203 ± 0,015 | 15,8 ± 0,173 |
| 7 | | Salt | 3,14 ± 1,684 | 9,067 ± 0,351 | 0,04 ± 0 | 0,77 ± 0,005 | 8,183 ± 0,006 | 15,533 ± 0,208 |
| 7 | | Combination | 5,323 ± 3,109 | 9,633 ± 0,058 | 0,04 ± 0 | 0,791 ± 0 | 8,22 ± 0,01 | 15,267 ± 0,058 |
| 8 | | Control | 4,987 ± 2,439 | 9,567 ± 0,306 | 0,03 ± 0 | 0,527 ± 0,003 | 8,19 ± 0,01 | 15,2 ± 0 |
| 8 | | Temperature | 5,58 ± 2,425 | 9,067 ± 0,208 | 0,03 ± 0 | 0,541 ± 0,001 | 8,24 ± 0,01 | 16,3 ± 0 |
| 8 | | Salt | 4,22 ± 0,636 | 8,667 ± 0,058 | 0,04 ± 0 | 0,818 ± 0,001 | 8,277 ± 0,031 | 16,1 ± 0 |
| 8 | | Combination | 6,047 ± 0,881 | 9,167 ± 0,115 | 0,04 ± 0 | 0,793 ± 0,002 | 8,173 ± 0,006 | 15,1 ± 0 |
| 9 | | Control | 5,143 ± 3,329 | 8,5 ± 0 | 0,03 ± 0 | 0,539 ± 0,001 | 8,303 ± 0,015 | 15,5 ± 0 |
| 9 | | Temperature | 4,617 ± 1,916 | 8,467 ± 0,058 | 0,03 ± 0 | 0,541 ± 0,001 | 8,24 ± 0,017 | 16,3 ± 0 |
| 9 | | Salt | 3,323 ± 2,064 | 9,2 ± 0,265 | 0,04 ± 0 | 0,818 ± 0,003 | 8,197 ± 0,006 | 16 ± 0 |
| 9 | | Combination | 6,22 ± 2,751 | 8,467 ± 0,058 | 0,04 ± 0 | 0,797 ± 0,001 | 8,243 ± 0,006 | 16 ± 0 |
| 10 | | Control | 4,837 ± 2,052 | 9,067 ± 0,153 | 0,03 ± 0 | 0,529 ± 0,002 | 8,38 ± 0,061 | 15,133 ± 0,058 |
| 10 | | Temperature | 6,563 ± 1,515 | 8,733 ± 0,058 | 0,03 ± 0 | 0,535 ± 0,001 | 8,247 ± 0,006 | 16,3 ± 0 |
| 10 | | Salt | 2,92 ± 0,426 | 8,5 ± 0,2 | 0,04 ± 0 | 0,82 ± 0,001 | 8,177 ± 0,006 | 15,9 ± 0 |
| 10 | | Combination | 4,127 ± 1,196 | 8,6 ± 0 | 0,04 ± 0 | 0,767 ± 0,004 | 8,25 ± 0,01 | 15,267 ± 0,115 |
| **Experiment 2 (October 2022)** | | | | | | | | |
| **Day** | | **Treatment** | **O_2_ Sediment in mg/l** | **O_2_ Water in mg/l** | **Salinity in %** | **Conductivity in mS/cm** | **pH** | **Temperature in °C** |
| 1 | | Control | 3,64 ± 0,476 | 9 ± 0,1 | 0,03 ± 0 | 0,642 ± 0,005 | 8,203 ± 0,021 | 16,433 ± 0,058 |
| 1 | | Temperature | 6,083 ± 0,327 | 9,1 ± 0,1 | 0,033 ± 0,006 | 0,669 ± 0 | 8,257 ± 0,006 | 15,9 ± 0 |
| 1 | | Salt | 6,81 ± 1,376 | 8,467 ± 0,306 | 0,03 ± 0 | 0,66 ± 0,002 | 8,167 ± 0,045 | 16 ± 0 |
| 1 | | Combination | 6,083 ± 0,327 | 9,1 ± 0,1 | 0,04 ± 0 | 0,661 ± 0,001 | 8,28 ± 0,02 | 16,367 ± 0,058 |
| 2 | | Control | 7,323 ± 0,451 | 8,667 ± 0,306 | 0,03 ± 0 | 0,633 ± 0,001 | 8,34 ± 0,01 | 16,333 ± 0,058 |
| 2 | | Temperature | 6,56 ± 1,532 | 8,933 ± 0,208 | 0,03 ± 0 | 0,649 ± 0,001 | 8,26 ± 0,01 | 15,933 ± 0,058 |
| 2 | | Salt | 5,75 ± 0,642 | 8,933 ± 0,058 | 0,27 ± 0 | 5,04 ± 0,01 | 8,183 ± 0,012 | 16,067 ± 0,058 |
| 2 | | Combination | 4,733 ± 1,405 | 8,8 ± 0,1 | 0,26 ± 0 | 4,927 ± 0,012 | 8,19 ± 0,01 | 18,533 ± 0,058 |
| 3 | | Control | 6,027 ± 2,183 | 8,867 ± 0,058 | 0,03 ± 0 | 0,636 ± 0,006 | 8,223 ± 0,029 | 16,9 ± 0 |
| 3 | | Temperature | 5,657 ± 1,938 | 8,433 ± 0,058 | 0,04 ± 0 | 0,682 ± 0,003 | 8,317 ± 0,006 | 20,6 ± 0 |
| 3 | | Salt | 4,047 ± 0,291 | 8,9 ± 0,2 | 0,27 ± 0 | 5,163 ± 0,012 | 8,347 ± 0,038 | 15,633 ± 0,058 |
| 3 | | Combination | 4,733 ± 1,405 | 8,8 ± 0,1 | 0,293 ± 0,006 | 5,497 ± 0,047 | 8,15 ± 0,026 | 19,9 ± 0 |
| 4 | | Control | 1,597 ± 0,47 | 8,933 ± 0,115 | 0,03 ± 0 | 0,63 ± 0,003 | 8,22 ± 0,01 | 16,233 ± 0,058 |
| 4 | | Temperature | 0,577 ± 0,335 | 8,133 ± 0,058 | 0,04 ± 0 | 0,677 ± 0,005 | 8,3 ± 0,01 | 20,7 ± 0 |
| 4 | | Salt | 1,123 ± 1,014 | 8,967 ± 0,153 | 0,27 ± 0 | 5,153 ± 0,015 | 8,203 ± 0,006 | 16 ± 0 |
| 4 | | Combination | 1,02 ± 0,668 | 8,2 ± 0,2 | 0,29 ± 0 | 5,497 ± 0,047 | 8,15 ± 0,026 | 19,9 ± 0 |
| 5 | | Control | 0,697 ± 0,489 | 8,867 ± 0,153 | 0,03 ± 0 | 0,639 ± 0,002 | 8,31 ± 0,026 | 16,533 ± 0,058 |
| 5 | | Temperature | 0,567 ± 0,809 | 8,033 ± 0,153 | 0,04 ± 0 | 0,679 ± 0,004 | 8,323 ± 0,006 | 20,467 ± 0,058 |
| 5 | | Salt | 0,3 ± 0,089 | 8,7 ± 0,1 | 0,28 ± 0 | 5,237 ± 0,006 | 8,197 ± 0,006 | 16,133 ± 0,058 |
| 5 | | Combination | 0,917 ± 1,381 | 8,133 ± 0,058 | 0,29 ± 0 | 5,53 ± 0,02 | 8,247 ± 0,006 | 20 ± 0 |
| 6 | | Control | 0,977 ± 0,598 | 9,033 ± 0,153 | 0,03 ± 0 | 0,633 ± 0,001 | 8,283 ± 0,012 | 16,033 ± 0,058 |
| 6 | | Temperature | 0,347 ± 0,453 | 9,5 ± 0,173 | 0,03 ± 0 | 0,637 ± 0,003 | 8,353 ± 0,057 | 15,2 ± 0 |
| 6 | | Salt | 0,587 ± 0,631 | 9,4 ± 0,265 | 0,18 ± 0 | 3,41 ± 0,01 | 8,31 ± 0,026 | 16 ± 0 |
| 6 | | Combination | 1,353 ± 1,446 | 9,067 ± 0,058 | 0,17 ± 0 | 3,293 ± 0,015 | 8,283 ± 0,006 | 15,2 ± 0 |
| 7 | | Control | 2,197 ± 1,522 | 8,2 ± 0,2 | 0,03 ± 0 | 0,66 ± 0,002 | 8,247 ± 0,006 | 15,7 ± 0 |
| 7 | | Temperature | 1,47 ± 1,067 | 8 ± 0 | 0,03 ± 0 | 0,651 ± 0,001 | 8,313 ± 0,006 | 15 ± 0 |
| 7 | | Salt | 2,873 ± 1,487 | 7,9 ± 0 | 0,05 ± 0 | 0,906 ± 0,005 | 8,343 ± 0,015 | 15,533 ± 0,058 |
| 7 | | Combination | 2,59 ± 0,725 | 7,767 ± 0,058 | 0,05 ± 0 | 0,911 ± 0,001 | 8,3 ± 0,062 | 15,8 ± 0 |
| 8 | | Control | 1,183 ± 0,754 | 8,433 ± 0,115 | 0,03 ± 0 | 0,64 ± 0,004 | 8,587 ± 0,05 | 15,8 ± 0,1 |
| 8 | | Temperature | 1,583 ± 0,714 | 9,367 ± 0,058 | 0,03 ± 0 | 0,653 ± 0,001 | 8,347 ± 0,006 | 15,167 ± 0,058 |
| 8 | | Salt | 1,037 ± 1,461 | 9,267 ± 0,208 | 0,05 ± 0 | 0,934 ± 0,003 | 8,327 ± 0,012 | 15,633 ± 0,058 |
| 8 | | Combination | 0,643 ± 0,584 | 9,6 ± 0,557 | 0,05 ± 0 | 0,913 ± 0,002 | 8,367 ± 0,015 | 15,767 ± 0,058 |
| 9 | | Control | 3,677 ± 1,598 | 8,867 ± 0,321 | 0,03 ± 0 | 0,643 ± 0,005 | 8,27 ± 0 | 15,533 ± 0,115 |
| 9 | | Temperature | 1,48 ± 0,864 | 9,567 ± 0,058 | 0,03 ± 0 | 0,629 ± 0,001 | 8,3 ± 0,01 | 14,9 ± 0 |
| 9 | | Salt | 1,233 ± 0,726 | 9,267 ± 0,058 | 0,05 ± 0 | 0,906 ± 0,004 | 8,317 ± 0,006 | 15,3 ± 0 |
| 9 | | Combination | 1,913 ± 2,033 | 9,067 ± 0,058 | 0,05 ± 0 | 0,909 ± 0,004 | 8,263 ± 0,012 | 15,6 ± 0 |
| 10 | | Control | 1,447 ± 0,754 | 8,867 ± 0,115 | 0,03 ± 0 | 0,628 ± 0,001 | 8,31 ± 0 | 15,6 ± 0 |
| 10 | | Temperature | 2,72 ± 1,015 | 9 ± 0,1 | 0,03 ± 0 | 0,636 ± 0,001 | 8,347 ± 0,015 | 15,7 ± 0 |
| 10 | | Salt | 0,177 ± 0,106 | 8,967 ± 0,153 | 0,05 ± 0 | 0,932 ± 0,003 | 8,323 ± 0,076 | 15,233 ± 0,058 |
| 10 | | Combination | 1,177 ± 0,072 | 8,867 ± 0,153 | 0,05 ± 0 | 0,915 ± 0 | 8,37 ± 0,046 | 15,6 ± 0,346 |
| **Experiment 3 (September 2023)** | | | | | | | | |
| **Day** | | **Treatment** | **O_2_ Sediment in mg/l** | **O_2_ Water in mg/l** | **Salinity in %** | **Conductivity in mS/cm** | **pH** | **Temperature in °C** |
| 1 | | Control | 2,68 ± 0,65 | 7,733 ± 0,058 | 0,03 ± 0 | 0,598 ± 0,001 | 8,35 ± 0,01 | 14,967 ± 0,058 |
| 1 | | Temperature | 3,067 ± 1,782 | 7,567 ± 0,153 | 0,03 ± 0 | 0,629 ± 0,002 | 8,287 ± 0,006 | 16,033 ± 0,058 |
| 1 | | Salt | 4,197 ± 2,778 | 8,333 ± 0,153 | 0,03 ± 0 | 0,609 ± 0,002 | 8,207 ± 0,049 | 15,633 ± 0,058 |
| 1 | | Combination | 5,147 ± 2,507 | 7,833 ± 0,252 | 0,03 ± 0 | 0,601 ± 0,001 | 8,303 ± 0,012 | 15,433 ± 0,058 |
| 2 | | Control | 2,79 ± 2,465 | 7,9 ± 0,173 | 0,03 ± 0 | 0,583 ± 0,001 | 8,337 ± 0,038 | 15,033 ± 0,058 |
| 2 | | Temperature | 1,9 ± 0,427 | 15,467 ± 0,231 | 0,03 ± 0 | 0,614 ± 0,004 | 8,203 ± 0,038 | 18,5 ± 0,1 |
| 2 | | Salt | 1,837 ± 1,507 | 16,067 ± 0,751 | 0,247 ± 0,006 | 4,733 ± 0,006 | 8,24 ± 0,026 | 16,5 ± 0 |
| 2 | | Combination | 3,857 ± 1,648 | 7,4 ± 0,346 | 0,197 ± 0,006 | 3,727 ± 0,006 | 8,257 ± 0,006 | 17,067 ± 0,058 |
| 3 | | Control | 2,38 ± 0,898 | 7,567 ± 0,058 | 0,03 ± 0 | 0,57 ± 0,003 | 8,323 ± 0,006 | 14,867 ± 0,115 |
| 3 | | Temperature | 3,903 ± 0,97 | 6,833 ± 0,058 | 0,033 ± 0,006 | 0,652 ± 0,002 | 8,397 ± 0,032 | 20 ± 0 |
| 3 | | Salt | 2,127 ± 1,334 | 7,2 ± 0,1 | 0,257 ± 0,006 | 5,013 ± 0,025 | 8,207 ± 0,059 | 16,2 ± 0 |
| 3 | | Combination | 4,137 ± 1,025 | 6,333 ± 0,058 | 0,277 ± 0,006 | 5,06 ± 0,017 | 8,23 ± 0,017 | 20,867 ± 0,058 |
| 4 | | Control | 2,62 ± 1,085 | 7,867 ± 0,569 | 0,03 ± 0 | 0,565 ± 0,002 | 8,173 ± 0,074 | 14,833 ± 0,058 |
| 4 | | Temperature | 1,99 ± 1,626 | 6,833 ± 0,153 | 0,04 ± 0 | 0,65 ± 0,005 | 8,39 ± 0,035 | 20,567 ± 0,058 |
| 4 | | Salt | 2,273 ± 2,605 | 6,633 ± 0,115 | 0,257 ± 0,006 | 5,03 ± 0,03 | 8,217 ± 0,071 | 15,833 ± 0,058 |
| 4 | | Combination | 4,133 ± 2,153 | 12,867 ± 0,306 | 0,277 ± 0,006 | 5,257 ± 0,006 | 8,223 ± 0,015 | 19,967 ± 0,058 |
| 5 | | Control | 1,207 ± 0,907 | 19,067 ± 1,021 | 0,03 ± 0 | 0,554 ± 0,003 | 8,297 ± 0,006 | 14,833 ± 0,058 |
| 5 | | Temperature | 2,88 ± 1,559 | 6,067 ± 0,058 | 0,03 ± 0 | 0,641 ± 0,002 | 8,427 ± 0,025 | 20 ± 0 |
| 5 | | Salt | 2,553 ± 1,76 | 5,667 ± 0,153 | 0,25 ± 0 | 4,887 ± 0,035 | 8,197 ± 0,051 | 16,533 ± 0,058 |
| 5 | | Combination | 1,903 ± 0,4 | 5,633 ± 0,058 | 0,283 ± 0,006 | 5,203 ± 0,021 | 8,233 ± 0,006 | 20,2 ± 0 |
| 6 | | Control | 2,573 ± 0,48 | 7,033 ± 0,115 | 0,03 ± 0 | 0,556 ± 0,003 | 8,3 ± 0 | 14,867 ± 0,058 |
| 6 | | Temperature | 5,267 ± 3,574 | 7,5 ± 0,173 | 0,03 ± 0 | 0,618 ± 0,002 | 8,3 ± 0 | 15,233 ± 0,058 |
| 6 | | Salt | 2,933 ± 1,467 | 7,333 ± 0,153 | 0,17 ± 0 | 3,22 ± 0,017 | 8,367 ± 0,058 | 12,9 ± 0 |
| 6 | | Combination | 3,677 ± 0,656 | 10,633 ± 0,231 | 0,177 ± 0,006 | 3,32 ± 0 | 8,3 ± 0 | 15,5 ± 0 |
| 7 | | Control | 3,413 ± 1,983 | 8,167 ± 0,208 | 0,03 ± 0 | 0,585 ± 0,002 | 8,333 ± 0,042 | 14,8 ± 0 |
| 7 | | Temperature | 1,41 ± 0,42 | 8,033 ± 0,153 | 0,03 ± 0 | 0,578 ± 0,007 | 8,277 ± 0,049 | 16 ± 0 |
| 7 | | Salt | 3,203 ± 0,826 | 7,5 ± 0,265 | 0,04 ± 0 | 0,814 ± 0,003 | 8,317 ± 0,006 | 16 ± 0 |
| 7 | | Combination | 6,44 ± 1,005 | 8 ± 0,265 | 0,04 ± 0 | 0,783 ± 0,002 | 8,27 ± 0,026 | 14,967 ± 0,058 |
| 8 | | Control | 2,33 ± 1,681 | 7,4 ± 0,2 | 0,03 ± 0 | 0,578 ± 0,001 | 8,317 ± 0,012 | 14,867 ± 0,115 |
| 8 | | Temperature | 0,937 ± 0,784 | 7,167 ± 0,208 | 0,03 ± 0 | 0,588 ± 0,002 | 8,32 ± 0,017 | 16,2 ± 0 |
| 8 | | Salt | 4,2 ± 2,797 | 7,7 ± 0,2 | 0,04 ± 0 | 0,834 ± 0,001 | 8,317 ± 0,006 | 16,233 ± 0,058 |
| 8 | | Combination | 3,643 ± 0,798 | 7,367 ± 0,208 | 0,04 ± 0 | 0,811 ± 0,001 | 8,32 ± 0,036 | 14,933 ± 0,058 |
| 9 | | Control | 3,34 ± 1,132 | 7,467 ± 0,208 | 0,03 ± 0 | 0,589 ± 0 | 8,333 ± 0,015 | 15,5 ± 0 |
| 9 | | Temperature | 4,607 ± 1,217 | 7,9 ± 0,1 | 0,03 ± 0 | 0,559 ± 0,032 | 8,313 ± 0,031 | 15,8 ± 0,1 |
| 9 | | Salt | 3,267 ± 1,072 | 7,867 ± 0,208 | 0,04 ± 0 | 0,836 ± 0,001 | 8,333 ± 0,015 | 16,033 ± 0,058 |
| 9 | | Combination | 4,42 ± 1,161 | 7,9 ± 0,3 | 0,04 ± 0 | 0,803 ± 0,001 | 8,26 ± 0,01 | 15,4 ± 0 |
| 10 | | Control | 0,65 ± 0,714 | 7,733 ± 0,231 | 0,03 ± 0 | 0,586 ± 0,003 | 8,473 ± 0,035 | 15,2 ± 0,1 |
| 10 | | Temperature | 1,88 ± 0,816 | 7,633 ± 0,153 | 0,03 ± 0 | 0,592 ± 0,001 | 8,37 ± 0,01 | 16,033 ± 0,058 |
| 10 | | Salt | 1,58 ± 1,235 | 7,367 ± 0,058 | 0,04 ± 0 | 0,839 ± 0,001 | 8,393 ± 0,015 | 16,133 ± 0,058 |
| 10 | | Combination | 2,097 ± 2,427 | 7,433 ± 0,058 | 0,04 ± 0 | 0,799 ± 0,001 | 8,353 ± 0,042 | 15,233 ± 0,058 |

**Table S2**. Reagents and cycling conditions of the first and the second PCR for 16S and 18S rRNA gene amplification. 20 cycles were used for the first PCR and 25 cycles were used for the second PCR.

| **PCR reagent** | **1^st^ PCR** | **2^nd^ PCR** |
| --- | --- | --- |
| Multiplex Master Mix | 5 µl | 5 µl |
| Forward Primer | 200 nM | 100 nM |
| Reverse Primer | 200 nM | 100 nM |
| DNA | 1 µl | 2 µl |
| Nuclease-free H_2_O | 3.6 µl | / |
| Coral Load | / | 1 µl |
| **Cycle conditions** |  |  |
| Initial denaturation | 5 min – 95 °C | 5 min – 95 °C |
| Denaturation | 30 s – 95 °C | 30 s – 95 °C |
| Annealing | 90 s - primer specific | 90 s – 61 °C |
| Elongation | 30s – 72 °C | 30s – 72 °C |
| Final Elongation | 10 min – 68 °C | 10 min – 68 °C |

**Table S3.** Summary of read and OTU Counts at different processing stages for 16S and 18S data in water and sediment samples

| **processing stage** | **Water_ 16S data** | **Water_18S data** |
| --- | --- | --- |
| after processing (Natrix2 pipeline) | 127,993,414 reads /  45,410 OTUs | 83,572,624 reads /  6,212 OTUs |
| after removal of negative controls | 82,423,921 reads /  45,340 OTUs | 66,639,984 reads /  6,210 OTUs |
| after noise filtering | 46,567,005 reads /  16,499 OTUs | 64,066,029 reads /  4,335 OTUs |
| **processing stage** | **Sediment_ 16S data** | **Sediment_18S data** |
| after processing (Natrix2 pipeline) | 148,205,825 reads /  40,969 OTUs | 74,499,619 reads /  8,603 OTUs |
| after removal of negative controls | 142,006,639 reads /  40,938 OTUs | 74,410,531 reads /  8,600 OTUs |
| after noise filtering | 134,448,300 reads /  16,267 OTUs | 63,140,374 reads /  5,182 OTUs |


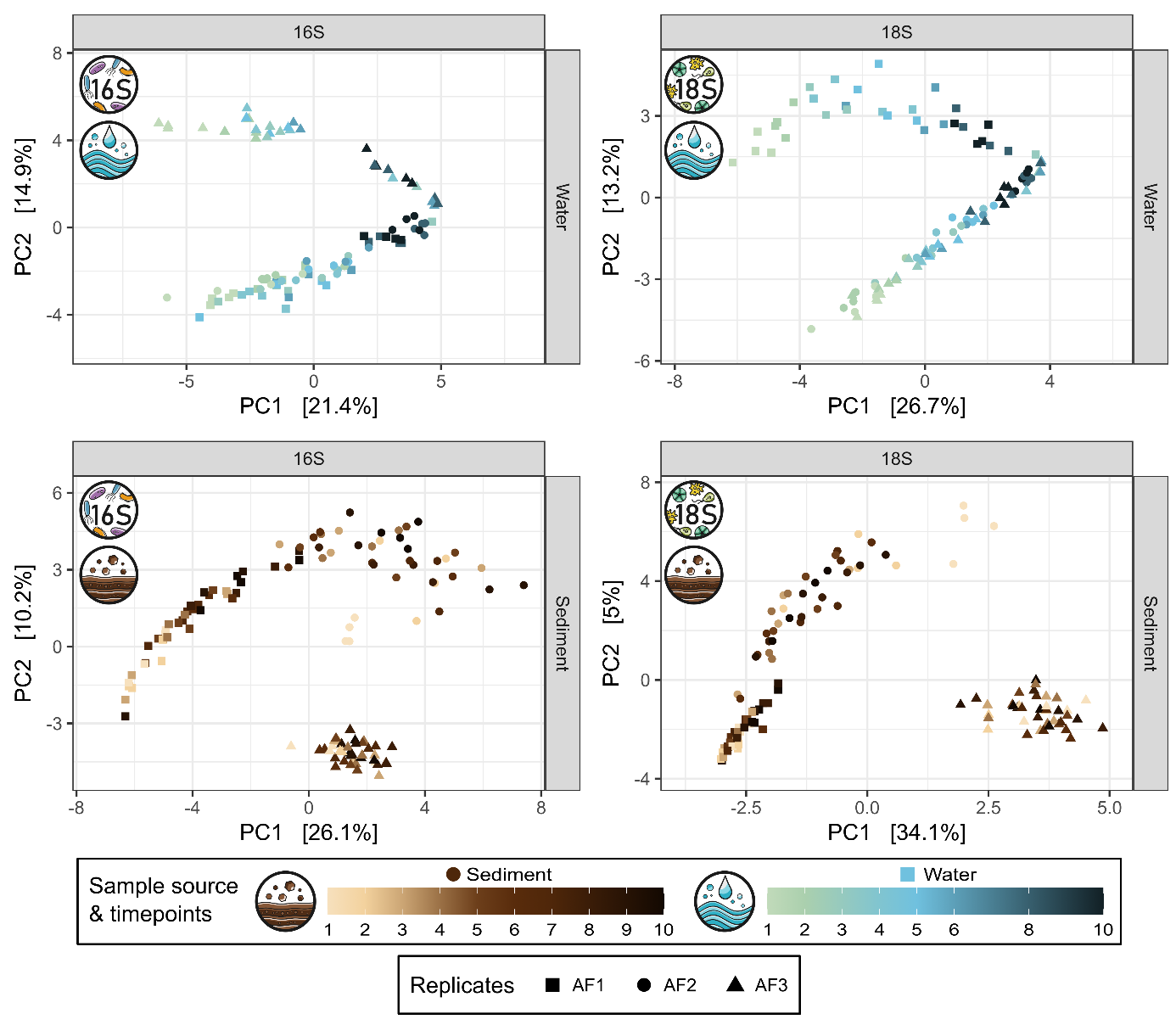


**Figure S1. Temporal effect and replicate variation of benthic and pelagic microbial communities.** Visualization via PCoA based on Bray-Curtis dissimilarity matrices for prokaryotic (left) and microeukaryotic community (right) in water (top) and sediment (bottom). Replicates are represented by different shapes, while the different sampling days are represented by a colour gradient.


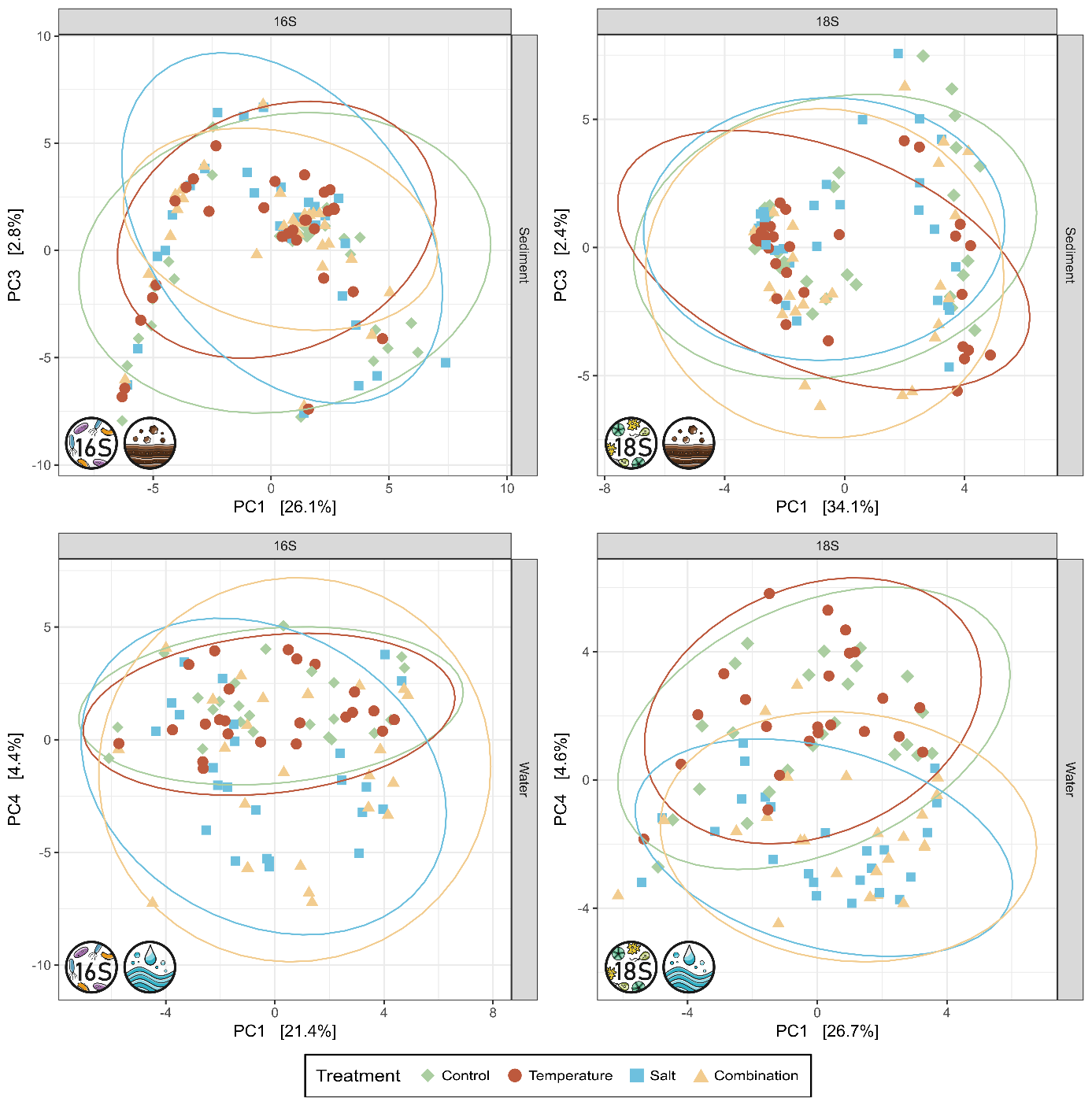


**Figure S2. Treatment effects of benthic and pelagic microbial communities.** Visualization via PCoA based on Bray-Curtis dissimilarity matrices for prokaryotic (left) and eukaryotic microbial (right) community in water (top) and sediment (bottom). The different treatments are visualized by different colors and shapes.


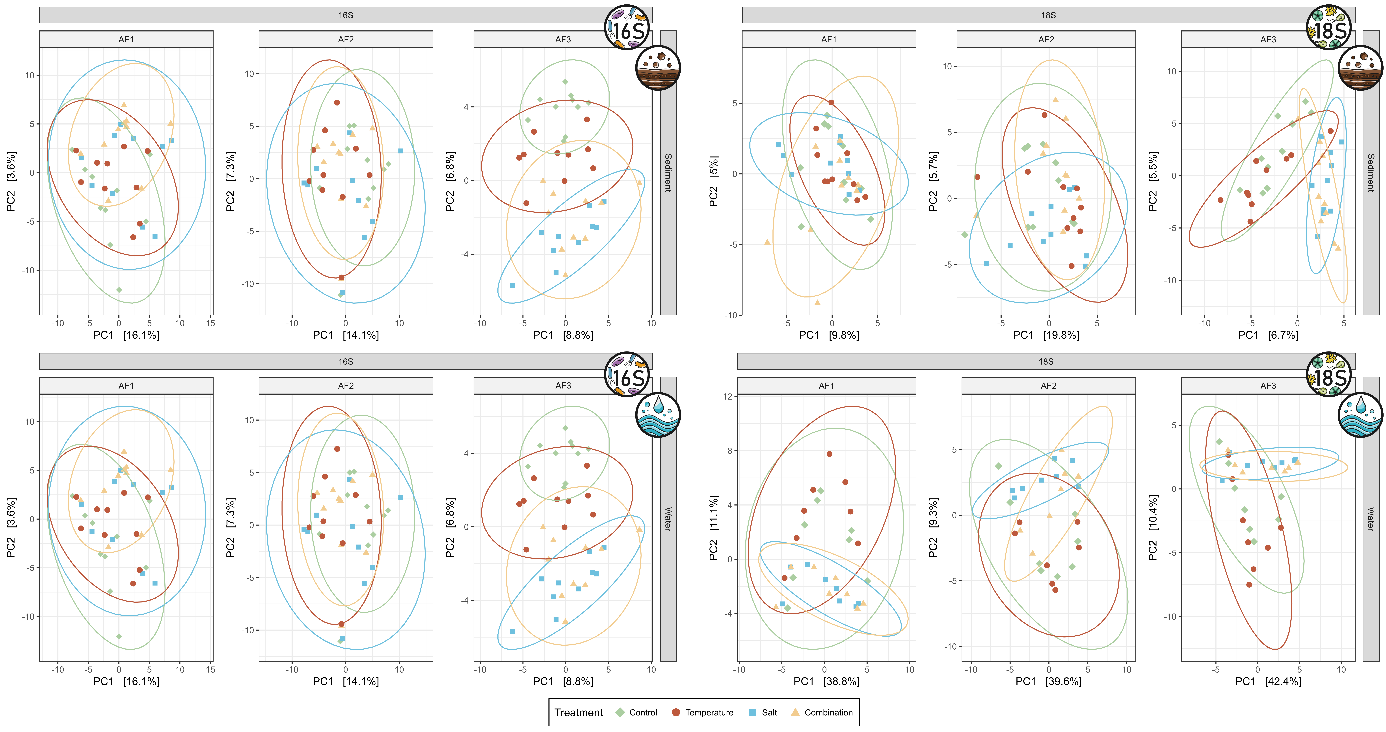


**Figure S3. Treatment effect on prokaryotic and microeukaryotic communities in water and sediment in each individual experiment.** Visualization based via PCoA and Bray-Curtis dissimilarity matrices for prokaryotic (left) and microeukaryotic community (right) in sediment (top) and water samples (bottom) from the individual experiments. PCoA plots depict treatment effect on axes 1 and 2 for AquaFlow experiment 1 (AF1; conducted in September 2022), AquaFlow experiment 2 (AF2; conducted in September 2022) and AquaFlow experiment 3 (AF3; conducted in September 2023). The data for each experiment was analysed separately.

**Table S4**. P-values from nested PERMANOVA testing the effect of treatments on microbial community composition while accounting for temporal variation for each individual experiment during the stressor phase. The data for each experiment was analysed separately.

| **Water** | | | | | | |
| --- | --- | --- | --- | --- | --- | --- |
|  | **Experiment 1** | | **Experiment 2** | | **Experiment 3** | |
|  | **16S** | **18S** | **16S** | **18S** | **16S** | **18S** |
| **Temperature** | 0.186 | 0.425 | 0.087 | 0.388 | 0.093 | 0.369 |
| **Salt** | 0.002 | 0.003 | 0.002 | 0.004 | 0.003 | 0.003 |
| **Combination** | 0.001 | 0.007 | 0.001 | 0.002 | 0.001 | 0.002 |
| **Sediment** | | | | | | |
|  | **Experiment 1** | | **Experiment 2** | | **Experiment 3** | |
|  | **16S** | **18S** | **16S** | **18S** | **16S** | **18S** |
| **Temperature** | 0.409 | 0.750 | 0.809 | 0.196 | 0.748 | 0.234 |
| **Salt** | 0.196 | 0.431 | 0.403 | 0.164 | 0.016 | 0.113 |
| **Combination** | 0.475 | 0.44 | 0.568 | 0.177 | 0.123 | 0.200 |


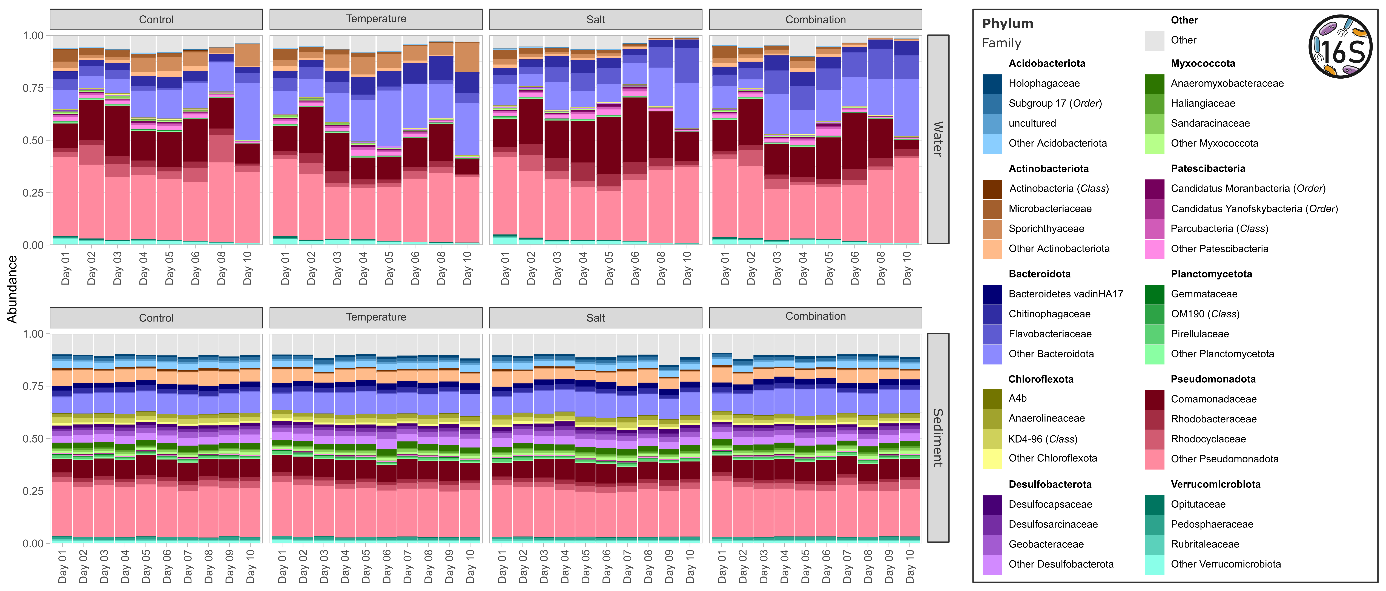


**Figure S4. Taxonomic composition of the prokaryotic communities in water (top) and sediment (bottom) exposed to different treatments.** Bar plots display the ten most abundant phyla and their three most abundant families. Families within the same phylum are represented by similar colors. 'Other' encompasses all additional taxa present in the samples. Samples were collected after acclimation but before stressor addition (D01), during the stressor phase (D02-D05), and after stressor removal (D06-D10).


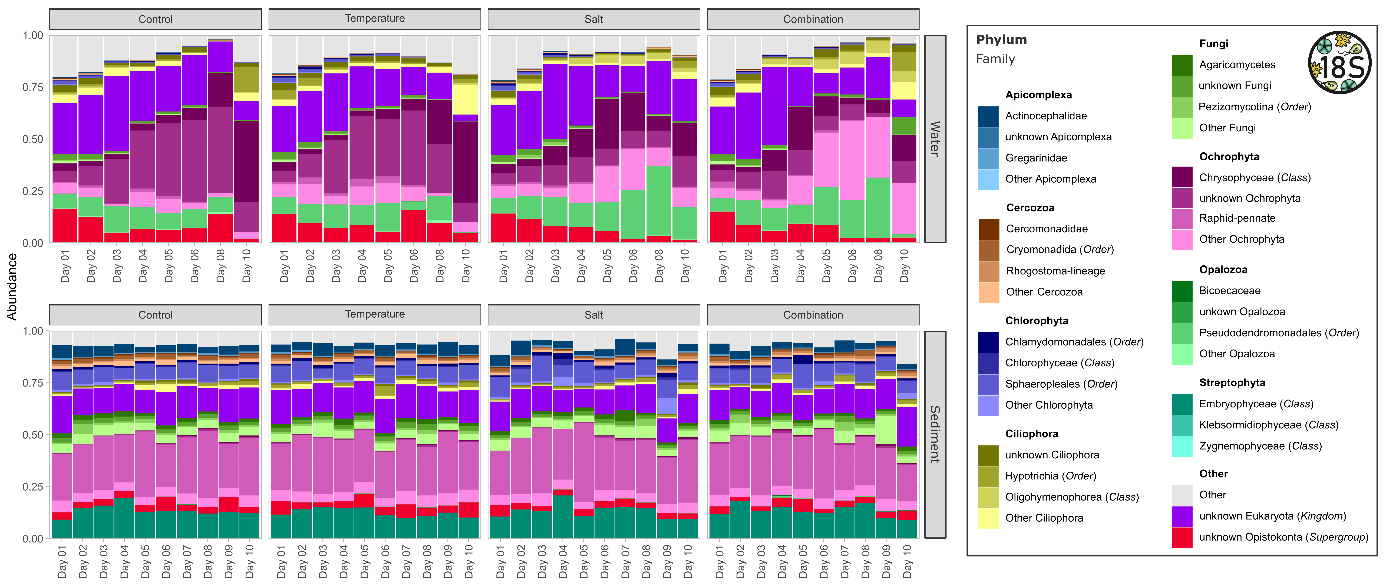


**Figure S5. Taxonomic composition of the microeukaryotic communities in water (top) and sediment (bottom) exposed to different treatments.** Bar plots display the ten most abundant phyla and their three most abundant families. Families within the same phylum are represented by similar colors. 'Other' encompasses all additional taxa present in the samples. Samples were collected after acclimation but before stressor addition (D01), during the stressor phase (D02-D05), and after stressor removal (D06-D10).


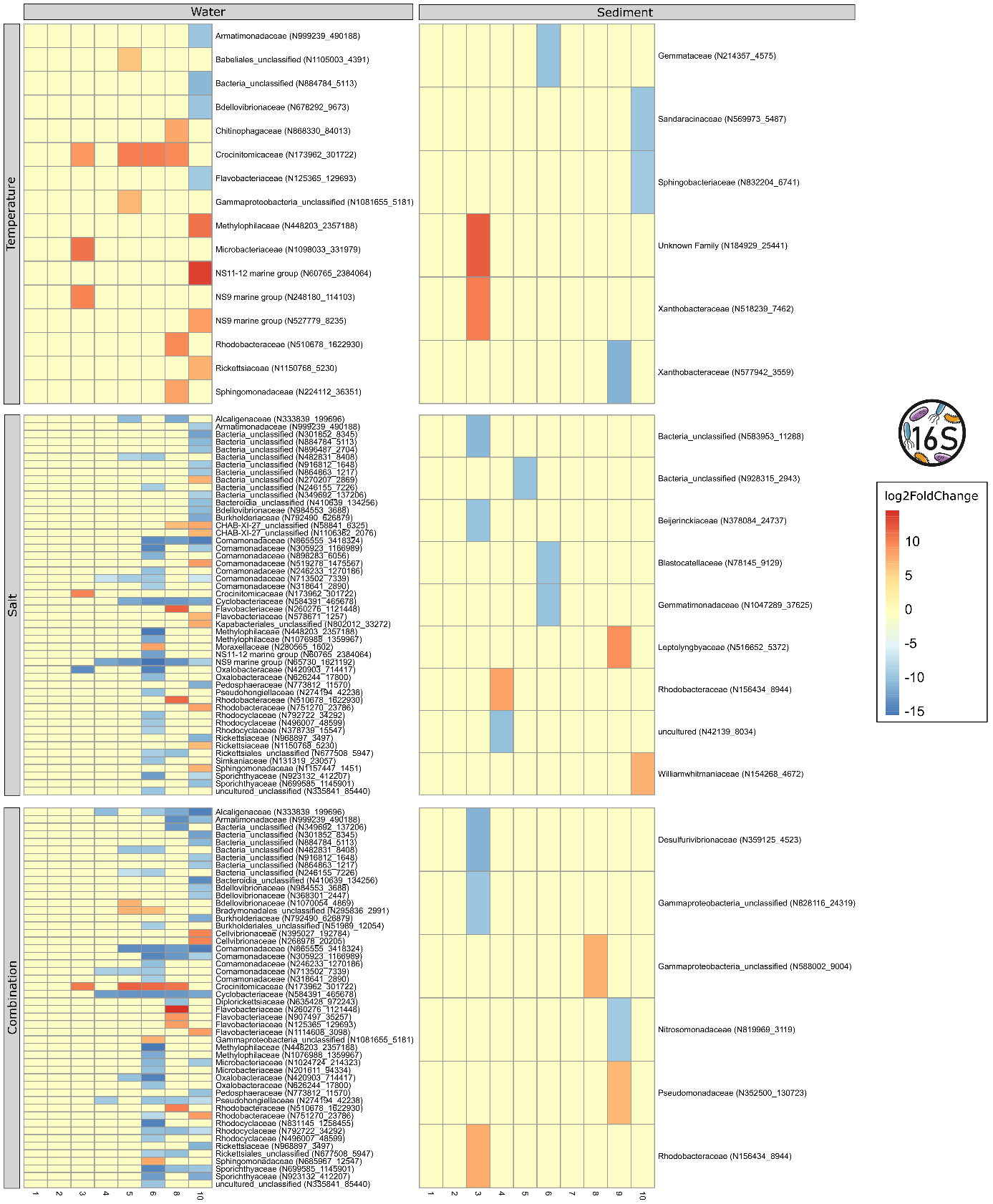


**Figure S6. Differentially abundant OTUs across treatments and time in the prokaryotic community.** Heatmaps visualizing temporal dynamics of the top 50 differentially abundant OTUs (ranked by maximum absolute log₂ fold change) per treatment and sample source across time points. Color intensity represents log₂ fold change relative to the control. OTU labels include taxonomic information at the family level.


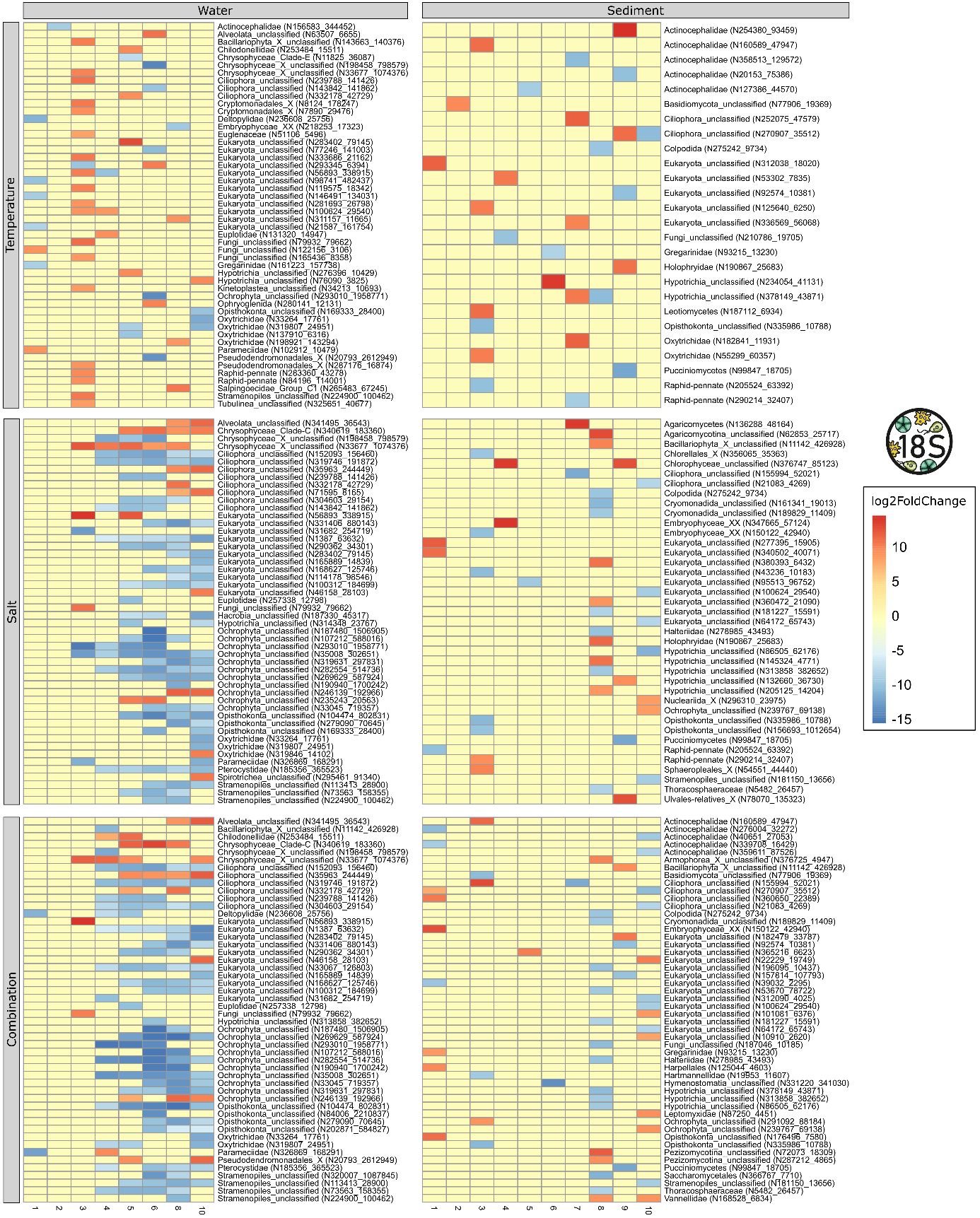


**Figure S7. Differentially abundant OTUs across treatments and time in the microeukaryotic community.** Heatmaps visualizing temporal dynamics of the top 50 differentially abundant OTUs (ranked by maximum absolute log₂ fold change) per treatment and sample source across time points. Color intensity represents log₂ fold change relative to the control. OTU labels include taxonomic information at the family level.
